# Tolerance to land-use changes through natural modulations of the plant microbiome

**DOI:** 10.1093/ismejo/wraf010

**Published:** 2025-01-21

**Authors:** Vincent Zieschank, Anne Muola, Stefan Janssen, Alexander Lach, Robert R Junker

**Affiliations:** Evolutionary Ecology of Plants, Department of Biology, University of Marburg, Karl-von-Frisch-Str. 8, Marburg 35043, Germany; Division of Biotechnology and Plant Health, Norwegian Institute of Bioeconomy Research, Holtvegen 66, Tromsø 9016, Norway; Algorithmic Bioinformatics, Justus-Liebig-University Gießen, Ludwigsplatz 13-15, Gießen 35392, Germany; Evolutionary Ecology of Plants, Department of Biology, University of Marburg, Karl-von-Frisch-Str. 8, Marburg 35043, Germany; Evolutionary Ecology of Plants, Department of Biology, University of Marburg, Karl-von-Frisch-Str. 8, Marburg 35043, Germany

**Keywords:** bacteria, fertilizer, fungi, microbiome, mowing, plant performance, plant phenotype

## Abstract

Land-use changes threaten ecosystems and are a major driver of species loss. Plants may adapt or migrate to resist global change, but this can lag behind rapid anthropogenic changes to the environment. Our data show that natural modulations of the microbiome of grassland plants in response to experimental land-use change in a common garden directly affect plant phenotype and performance, thus increasing plant tolerance. In contrast, direct effects of fertilizer application and mowing on plant phenotypes were less strong. Land-use intensity-specific microbiomes caused clearly distinguishable plant phenotypes also in a laboratory experiment using gnotobiotic strawberry plants in absence of environmental variation. Therefore, natural modulations of the plant microbiome may be key to species persistence and ecosystem stability. We argue that a prerequisite for this microbiome-mediated tolerance is the availability of diverse local sources of microorganisms facilitating rapid modulations in response to change. Thus, conservation efforts must protect microbial diversity, which can help mitigate the effects of global change and facilitate environmental and human health.

## Introduction

Land-use changes such as mowing or fertilizer application in grasslands are a major driver of species loss and responsible for alterations in community structure, with ensuing consequences for ecosystem functioning and human well-being [1, 2]. Plant species are increasingly challenged to cope with these environmental alterations. Migration to more suitable habitats or adaptations to novel conditions are frequently discussed mechanisms to avoid extinction [3, 4], which may, however, lag behind rapid anthropogenic alterations of environmental conditions [5]. Natural modulations of plant-associated microbial communities in response to environmental stresses can represent an alternative and possibly faster way for plants to cope with alterations in land-use and other components of global change [6–9]. In fact, ecological and evolutionary microbiome responses can easily keep pace with environmental changes [10] and land-use history, intensity, and agricultural practices have been shown to affect the diversity and composition of bacterial and fungal communities in the soil or associated with plants [11–15]. These rapid responses of plant-associated microbiomes may then, in turn, have positive effects on plant phenotype and productivity, increasing plant species’ tolerance to changes [16, 17]. As a consequence, plants may invariantly perform well despite being challenged by stressors [18–20]. Accordingly, inoculation experiments in the context of climate change showed that a preadapted microbiome can increase survival rates of seedlings and promote plant growth under environmental stress [9, 20, 21]. Positive microbial effects on plant species could even promote species richness and productivity in plant communities [12, 14, 22]. Therefore, spontaneous natural changes in plant-associated microbiomes may be equivalent to recent advances in microbiome engineering, which is used to promote plant fitness and support plant tolerance to rapid changes of the environment [23].

For grasslands in particular, land degradation and changes in land-use intensity such as mowing or fertilizer application were identified as the main causes of the decline in species diversity, the disruption of interactions between species, and the loss of ecological functions [2, 24–26]. Different components of land-use (e.g. mowing or fertilizer application) have been shown to affect plant and microbial diversity and composition differently [13, 27–29]. Although some plant species and other taxa benefit from an increase in land-use intensity, many plant species decline under intensive land-use and are eventually displaced from their original habitat [2]. Plants in natural and agricultural systems may benefit from microbes, which can help plants individuals to resist abiotic stresses or protect them against diseases and herbivores [20, 21, 30, 31]. Both individual strains of microbes as well as diverse consortia can affect plant traits, plant interactions, and ecosystem processes and functions [22, 32–34], but inoculated consortia usually outperform individual strains [35].

Based on the previous knowledge about the vulnerability of grassland species [27, 36] and the responses of bacteria and fungi to land-use changes [13, 37], we tested whether natural rapid modulations of the plant microbiome have the potential to mediate land-use effects on plant phenotype and performance, and thus might represent an alternative to the “adapt or migrate” strategies. In a common garden experiment, grass sods from different regions in Germany were subjected to different mowing and fertilizer regimes [29] to test the effects of land-use treatments on morphological and physiological features of plant communities. Additionally, the sods were complemented with *Fragaria vesca* plants as phytometers to also test effects of land-use treatments on *F. vesca* phenotype and performance, as well as the diversity and composition of the bacterial and fungal communities associated with *F. vesca*. Such experiments may reveal strong microbial contributions to land-use effects on plant phenotype and performance [9, 21, 22], but clear experimental evidence for direct effects of the plant microbiome modulated by global change on plant responses requires additional, more controlled laboratory experiments. Therefore, we inoculated *F. vesca* plants grown from surface sterilized seeds in germ-free containers with the whole microbiome extracted from field-grown phytometers, testing microbial effects on the plant phenotype under gnotobiotic conditions in the absence of environmental variation.

With these common garden and laboratory experiments, we tested a set of hypotheses that specifically address the prerequisites underlying the rationale of microbiome-assisted plant tolerance to land-use changes: (i) microbial diversity and composition as well as single plant phenotype and plant community features rapidly respond to major components of land-use, mowing and fertilization; (ii) effects of the microbiome on plant phenotype and performance are as strong or even stronger than effects of mowing and fertilization; (iii) if rapid alterations in the plant’s microbiome increase the tolerance of plants to land-use changes, plant performance should be relatively stable across land-use treatments; (iv) microbial effects are also measurable in the absence of other biotic or abiotic influences (as tested in the lab experiments). By verifying these hypotheses, we show that rapid natural modulations of the plant microbiome may increase plant tolerance to land-use changes and therefore may also buffer grasslands against stressors of global change.

## Material and methods

### Plant communities

The project is part of the Biodiversity Exploratories, a large-scale research network with three long-term research sites (“Exploratories”) in Germany at Schwäbische Alb, Hainich, and Schorfheide-Chorin, where land-use and biodiversity of 50 grassland plots per region have been continuously recorded [38]. In 2020, we established a common-garden (“EXClAvE”) to track effects of land-use by reducing environmental heterogeneity to a minimum. In each region, we selected 13 plots that evenly cover the range of land-use intensity (measured as land-use index LUI, see [39]). On each pre-selected plot, we collected grass sods the size of 1m^2^ and 10 cm depth in April and May 2020. The sods were split into four parts of 50x50cm and each part was subsequently randomly assigned to one of four experimental land-use treatments: “00” (= mowing once per year), “M0” (= mowing twice per year), “0F” (= mowing once per year and fertilizer addition) and “MF” (= mowing twice per year and fertilizing). Together with 12 sods made from local steam-sterilized soil to monitor spontaneous plant establishment from the surroundings, the common garden consists of 168 sods/plant communities (for more information see [29]). Land-use treatments first started in mid-July 2020. Plant communities were mown in early summer and autumn each year and fertilization (99 kg N ha^−1^ year^−1^) was done right after the first mowing, respectively (see [29]).

### Digital phenotyping

For data collection, we customized an automated plant phenotyping system (PlantEye F500, Phenospex, Heerlen, The Netherlands) for its mobile application in the field (described in [29]). By scanning whole plant communities, we gather, within seconds and non-invasively, multispectral and physiological information while simultaneously capturing the 3-dimensional structure of the vegetation. The scans are processed with the built-in software HortControl (Phenospex, Heerlen, The Netherlands) that provides multiple morphological and physiological parameters [29]. The accuracy of this method in tracking plant community responses to mowing and fertilization was demonstrated in [29]. For this study we excluded the parameter “maximum height” from all analyses because of its unsuitability for scans of whole communities. The scans of plant communities used in this study were recorded in August 2021, according to the time at which the *F. vesca* plants were removed for the analyses after 3 months of growth in the communities.

### *Fragaria* field experiment

We used *F. vesca* plants (accession: PI616612, USDA National Clonal Germplasm Repository, Corvallis, US) as phytometers in the common garden because this species has been recorded at the grassland plots of the Biodiversity Exploratories but does not naturally occur on any of the chosen plots sampled for the common garden. *F. vesca* is an herbaceous perennial plant that occurs throughout the Northern Hemisphere [40] and can grow in various habitats such as grasslands, roadsides, open forests, and forest and farmland edges [41, 42]. *F. vesca* flowers are hermaphrodite and self-compatible to various degrees [43, 44]. *F. vesca* is insect pollinated and reproducing both sexually and clonally through formation of aboveground runners [42, 45]. To prepare the experiment, we grew *n* = 380 individuals of a clone of *F. vesca* in a greenhouse. Based on morphological and physiological parameters from digital phenotyping [29], we selected a subset of *n* = 168 plants with a homogenous phenotype. Immediately prior to transferring the plants to the common garden, all *F. vesca* plants were digitally phenotyped. Each plant was scanned from two opposite sides, which were marked on the pot for repeated scans at the end of the experiment. The mean value of the two scans was used as dependent variable. We planted one individual of *F. vesca* into each sod of the common garden in May 2021. The plants remained in their pots with the original soil in order to exclude soil and nutrient effects of the sods on plant growth and performance. After a one-week acclimatization period, *F. vesca* plants received the same land-use treatment as the surrounding plant community: “00” (= no treatment), “M0” (= mowing), “0F” (= fertilizer addition) and “MF” (= mowing and fertilizing). For the mowing treatment, plants were cut at ~2 cm above ground and the fertilizer quantity was adjusted to match the fertilization of the sods (99 kg N ha^−1^ year^−1^). After three months, we recorded herbivory (visual assessment of % of leaf damage after online training using the ZAX Herbivory Trainer [46], which improves a correct assessment of the feeding damage to leaves by repeatedly estimating feeding damage of virtual leaves), fruit set (number of fruits and flowers) and vegetative reproduction (number of stolons and offshoots) as proxies for performance. *F. vesca* plants were scanned again from the previously marked sides and one leaf and root sample was taken from each plant for high-throughput sequencing. To account for small differences in the phytometers’ phenotype prior to the experiment, we used the differences in phenotypic parameters between the scan after and before the experiment as dependent variable in all further statistical analyses.

### Microbiome analysis

Leaf and root samples of *F. vesca* phyotmeters were collected using sterilized forceps (dipped into 70% ethanol and flamed) to avoid contamination using established protocols [13, 34, 47–49]. Samples were directly transferred to ZR BashingBead Lysis tubes containing 750 μL of ZymoBIOMICS lysis solution (Zymo-BIOMICS DNA Miniprep Kit, Zymo Research, Irvine, CA). ZR BashingBead Lysis tubes were sonicated for 7 minutes right after the collection of microbial samples to detach microorganisms from the surfaces. Leaf and root tissue was subsequently removed from the tubes under a sterile bench with sterile forceps to reduce the amount of plant DNA in the samples. All microbial samples were then shaken using a ball mill for 9 minutes with a frequency of 30.0 s^−1^. Microbial DNA was isolated using the ZymoBIOMICS DNA Miniprep Kit following the manufacturers’ instructions. Amplicon sequencing of isolated DNA samples was performed by Eurofins Genomics using the company’s standard procedure “InView - Microbiome Profiling 3.0 with MiSeq” (Ebersberg, Germany). The V3-V4 region of the 16S rRNA (Primer: Fwd: TACGGGAGGCAGCAG, [50]; Rev: CCAGGGTATCTAATCC, [51]) and the ITS region (Primer: Fwd: GCATCGATGAAGAACGCAGC; Rev: TCCTCCGCTTATTGATATGC, [52]) of leaf and root microbiome samples were amplified and sequenced with Illumina MiSeq to characterize bacterial and fungal communities, respectively. Microbiome profiling of isolated DNA was performed on the Qiita web platform [53]. Prior to upload into Qiita, sequences have been clipped free of sequencing adapters (cutadapt v 4.2 with primer sequences CCTACGGGNGGCWGCAG and GACTACHVGGGTATCTAATCC) and raw sequences of the bacterial and fungal data were demultiplexed and quality filtered using the q2-demux plugin followed by denoising with DADA2 (via q2-dada2) [54]. Forward and reverse reads were joined and trim length was 150 bp for bacterial and 250 bp for fungal sequences. Taxonomy was assigned to bacterial ASVs using the q2-feature-classifier [55] against the SILVA 138 99% OTUs reference sequences [56, 57], and to fungal ASVs using the classify-sklearn naïve Bayes taxonomy classifier against the UNITE 97% Version 10.0 reference sequences [58]. Archaeal and eukaryotic sequences were excluded from further analysis, and low frequency taxa in the datasets were removed as well (ASVs that occurred in less than 10% of the samples). On average, we received the following sequencing coverage: leaf-associated bacteria: 65,109.82 ± 17,171.97 mean ± SD (min 19,471; max 65,109); root-associated bacteria: 59,066.66 ± 15,376.9 (min 12,753; max 89,366); leaf-associated fungi: 60,189.33 ± 21,245.73 (min 2,139; max 195,057); root-associated fungi: 59,402.9 ± 46,459.46 (min 11,383; max 580,783). In total, we detected *n* = 2,765 bacterial ASVs and *n* = 791 fungal ASVs. The raw sequences of high-throughput 16S and ITS amplicon sequencing are available at the European Nucleotide Archive ENA under the project accession PRJEB52951 (https://www.ebi.ac.uk/ena/browser/view/PRJEB52951; sequences not adapter trimmed). Prior to the statistical analysis of microbial communities, we performed a cumulative sum scaling (CSS) normalization (R package metagenomeSeq v1.28.2 [59]) on the count data to account for differences in sequencing depth among samples. CSS offers superior normalization in marker-gene microbial surveys by effectively addressing biases from uneven sequencing depth, thereby enabling more accurate differentiation of samples based on phenotypic similarity [60]. Furthermore, CSS also minimizes the influence of reads with high abundances and thus positively affects the distribution of the data [61].

### *Fragaria* lab experiment

In order to transplant the full microbiome of the phytometers to individuals of the *F. vesca* clone, we first removed soil from roots and then placed the plants into plastic bags filled with 225 ml sterile water and shook them in a pulsifier (Microgen Bioproducts Ltd, Camberley, Surrey, UK) for 60 seconds (following the user manual). 1 ml of each extract was stored in a freezer (−80°C) after adding 0.5 ml Glycerin for future use in the follow-up inoculation experiment. Seeds from the same *F. vesca* clone were sterilized by sequential exposure to 70% ethanol (10 min) and a 12% sodium hypochlorite solution (5 min) and four subsequent washes with sterile water (following the protocol of [62]). After 24 hours in the fridge, sterilized seeds were put individually into pre-autoclaved (121°C, 20 min, 1.1 bar) microcosm containers with tightly closing lids containing a filter that allows gas exchange but no passage of microorganisms (SacO2 O118/120 + OD118/80, SacO2 NV, Veldeken 29, 9850 Deinze, Belgium). Each box was prepared with 90 g clay (Diamond Pro Calcined Clay drying agent, Diamond Pro, 1112 E. Copeland Rd, Ste 500, Arlington, TX 76011, Texas, USA) that has been washed repeatedly before drying and sterilizing it in a drying cabinet at 200°C for 24 h. For nutrient and water supply we added autoclaved 50 ml plant agar (5.5 g L^−1^, Duchefa Biochemie B.V., 2003 RV Haarlem, The Netherlands) containing 1.1 g L^−1^ Murashige & Skoog Medium including vitamins (Duchefa Biochemie B.V., 2003 RV Haarlem, The Netherlands) to each container. Two weeks after the emergence of the primary leaf, the plants were inoculated with the stored whole microbiome extract by pipetting each 10 μl on three leaves of the plant. Per donor plant, two receiver plants were inoculated, resulting in *n* = 332 inoculated plants. Plants grew for three months in a climate chamber at 20°C and a 15 h/9 h light/dark cycle and were finally digitally phenotyped. Unfortunately, the scanner had an error during these scans, reducing the sample size from *n* = 166 to *n* = 95, which did, however, not lead to a bias towards or against certain treatment(s).

### Statistical analyses

We used structural equation models (SEMs) that offer a statistical framework for examining various processes that potentially influence system characteristics. SEMs are particularly well-suited to assess complex hypotheses with multiple pathways or levels of interaction [63]. Specifically, we applied SEMs to (i) test the effect of mowing and fertilizing treatments on plant community features, individual plant phenotype, the plant-associated microbiome diversity and composition, and plant performance; (ii) to investigate the role of the microbiome in mediating effects of mowing and fertilizing on plant phenotype and performance; and (iii) to compare the relative effect sizes of the microbiome and the land-use treatments on plant phenotype and performance. Accordingly, we defined fertilizer and mowing treatments as exogenous variables that may directly affect plant community features, *F. vesca* phenotype and performance, and the plant-associated microbiome. We further tested for effects between the dependent variables to explore potential mechanisms that explain plant phenotype and performance. We built separate models with identical structure for microbiome diversity and microbiome composition. Pathways represent direct effects of mowing and fertilization on the plant community, *F. vesca* phenotype, *F. vesca* performance, and microbiome composition or microbiome diversity. We also included direct effects of the plant community on *F. vesca* phenotype and microbiome composition/diversity, of *F. vesca* phenotype on *F. vesca* performance, and of microbiome composition/diversity on *F. vesca* performance. Both exogenous variables in the model (mowing and fertilizing) were used as categorical binary predictors (fertilized / not fertilized; mown once / mown twice a year). Since the fixed range of categorical variables disrupts the presumed relationship between standard deviation and range of a predictor, thus interfering with the standardization of estimates within a model, we recalculated standardized estimates for relevant paths following the tutorial of Grace [64], using defined-difference standardization where the defined difference is the known range of values [65].

For the multivariate variables plant community features, *Fragaria* phenotype, *Fragaria* performance and microbiome composition in the SEM, we used principal components (PC1) as univariate representatives. PCA for plant community features and *Fragaria* phenotype included 13 parameters from digital phenotyping. *Fragaria* performance comprised inverse herbivory (% of undamaged leaf area), number of flowers, fruits, stolons, and offshoots. For microbiome composition, we combined leaf and root ASV abundance data for bacteria and fungi, respectively, then merged bacterial and fungal ASVs for the final whole microbiome PCA. In order to be able to test for microbiome diversity (and not only for bacteria and fungi diversity separately), we used a multidiversity index (according to a previously published method [66]) that puts equal weights to diversities of different taxa despite differences in total number of species between these taxa. First, we calculated Shannon diversity indices separately for bacteria and fungi in each sample from the combined leaf and root ASV abundance data (using non-normalized data) by rarefying the data to the minimum number of reads available in the samples (iterations: *n* = 999). Second, we separately ranked samples by increasing diversity for bacteria and fungi and then calculated the mean rank ((rank_bacteria_ + rank_fungi_) / 2) per sample to get a cumulative microbiome diversity. For better interpretability we scaled mean ranks between zero and one. In that way, we gave the same weight to bacteria and fungi despite deviations in the absolute values of Shannon diversity [66]. To account for the setup of the common garden, we tested for the presence of residual spatial autocorrelation in the estimated path models by calculating spatial neighbor matrices (k = 8) based on plant community coordinates and estimating Moran’s I for the case-wise residuals of the exogenous variables of both models using the package *spdep* (v1.2–7 [67]). Significant autocorrelation was only found for the residuals of the plant community variable in both models (*P* < 0.001, see Supplementary Table 1). Following a tutorial by Jim Grace (https://bit.ly/44kJ9gt, based on an approach from: Bivand, Pebesma [68]) we estimated the real effective sample size (*n* = 128), then adjusted standard errors accordingly and recalculated *P* values for the model parameters affected by the autocorrelation. Both models were estimated using the R-package *lavaan* (v0.6–12 [69]).

We performed further analysis for a closer insight into the significant relationships between variables in the SEM. For a multivariate assessment of the effects of mowing and fertilizing on plant community, *Fragaria* phenotype, *Fragaria* performance and microbiome composition, we performed distance-based redundancy analyses using Bray-Curtis distances followed by a permutation test under reduced model with subsequent analysis of variance using functions *capscale* (R package vegan v2.6–2 [70]) and *anova* (R base package stats 4.2.0 [71]) with mowing and fertilizing separately as factors. Furthermore, we performed an *anova* (R base package stats 4.2.0 [71]) with mowing and fertilizer as factors to explore the response of microbial diversity to land-use components and to look for treatment-associated microbiome-induced changes in the phenotype of previously inoculated lab plants. We used the default workflow steps implemented in the R package *DESeq2*, a tool for differential expression analysis, to trace significant changes triggered by mowing and fertilization in bacteria and fungi ASVs [72]. The strength of effects is measured as log2 fold change, which means the land-use component induced a multiplicative change of 2^−1^ = 0.5 in abundance of the observed ASV compared to plants that did not receive this treatment.

## Results

### Plants and microbiomes rapidly respond to land-use treatments

**Figure 1:**
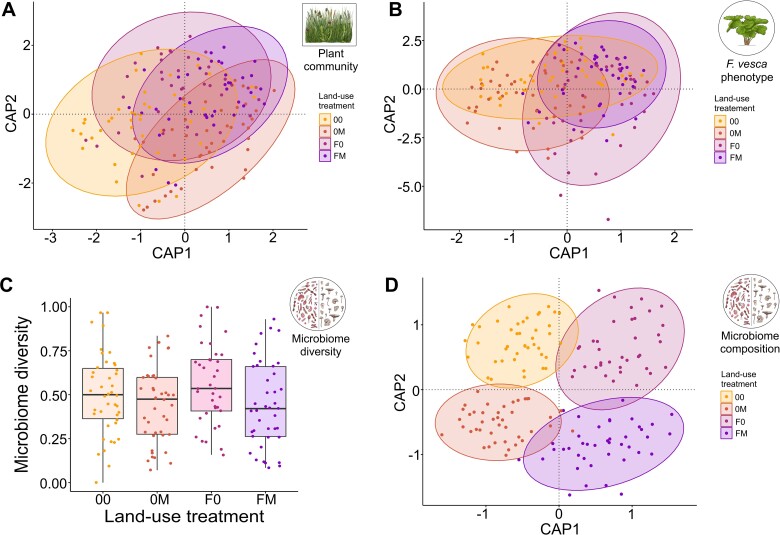
Land-use effects on plant communities features, the phenotype of plant individuals and microbiomes in a common garden experiment. Plant community features (A) and the *F. vesca* phenotype (B) were assessed by 13 morphological and physiological parameters using a 3D plant scanner and both clearly responded to land-use treatments. Microbiome diversity (C) and composition (D) were analyzed based on 16S rRNA (bacteria) and ITS2 (fungi) amplicon sequencing and both, diversity and composition, responded to land-use treatments. Each circle represents one sample, land-use treatment is color-coded; “00” (= mowing once per year), “0 M” (= mowing twice per year), “0F” (= mowing once per year and fertilizer addition) and “MF” (= mowing twice per year and fertilizing). Statistical results are reported in the text and supplementary material (Supplementary Tables 1 & 10).

The plant communities of the transplanted grass sods that each contained one individual of a *F. vesca* clone were subjected to one of four land-use treatments: “00” (= mowing once per year), “M0” (= mowing twice per year), “0F” (= mowing once per year and fertilizer addition) and “MF” (= mowing twice per year and fertilizing). Within the duration of the experiment, *F. vesca* plants received the following corresponding treatments: “00” (= no treatment), “M0” (= mowing), “0F” (= fertilizer addition) and “MF” (= mowing and fertilizing). Three months after *F. vesca* individuals were placed in the common garden, we assessed morphological and physiological features of the plant communities as well as *F. vesca* individuals by scanning them with a customized plant phenotyping system (PlantEye F500, Phenospex, Heerlen, The Netherlands). The plant scanner was originally constructed to scan plant individuals, but also provides meaningful information on whole plant communities [29]. A total of *n* = 162 plant communities and *F. vesca* plants were included in the final analyses. In accordance with our first hypothesis, we found strong effects of mowing and fertilizer addition on plant communities (Constrained Analysis of Principal coordinates (CAP) based on Bray–Curtis distances followed by permutation test for capscale under reduced model; mowing: *F*_1,158_ = 7.87, *P* < 0.001; fertilizer: *F*_1,158_ = 7.27, *P* < 0.001; mowing × fertilizer: *F*_1,158_ = 1.96, *P* = 0.029; Fig. 1A). Individual community features differed between treatments (see Supplementary Fig. 1).

**Figure 2:**
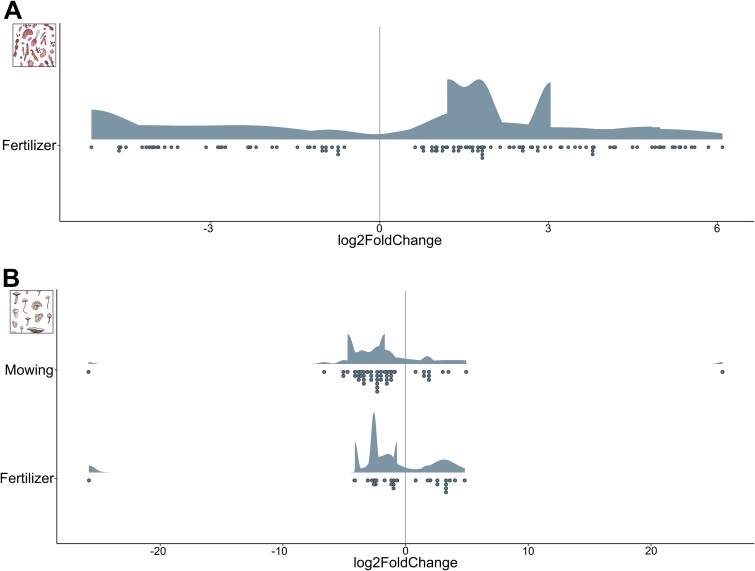
Land-use effects on individual bacterial (A) and fungal (B) ASVs. ASVs that significantly responded to mowing or fertilizer application have been identified using the R package *DESeq2.* log2FoldChange gives an estimate for effect size. A detailed list of the taxonomy of significant ASVs can be found in the supplementary material (Supplementary Tables 6-8).

Likewise, *F. vesca* plant individuals also clearly responded to land-use treatments by alterations in morphological and physiological features (CAP: fertilizer: *F*_1,158_ = 10.53, *P* < 0.001; mowing × fertilizer: *F*_1,158_ = 1.96, *P* = 0.029; Fig. 1B). All morphological traits, including digital biomass, height, leaf angle, leaf area, leaf area index, leaf area projected, leaf inclination and light penetration depth differed significantly between fertilized and unfertilized plants (see Supplementary Table 3 and Supplementary Fig. 2 for detailed results). The only physiological parameter significantly influenced by fertilization was greenness. Mown and unmown plant individuals significantly differed in the three leaf area-associated indices leaf area, leaf area index and leaf area projected (see Supplementary Table 3 and Supplementary Fig. 3 for detailed results). Furthermore, bacterial and fungal communities that are associated with *F. vesca* roots and leaves (analyzed via high-throughput 16S rRNA and ITS amplicon sequencing) strongly responded to land-use treatments, too. Microbial multi-diversity responded negatively to mowing (ANOVA: *F*_1,158_ = 6.27, *P* = 0.013; Fig. 1C, see also Supplementary Table 4 for results on bacteria and fungi associated to leaves and roots separately) and land-use effects on microbiome composition were even more pronounced (CAP based on Bray–Curtis; mowing: *F*_1,158_ = 1.73, *P* < 0.001; fertilizer: *F*_1,158_ = 3.39, *P* < 0.001; Fig. 1D, see also Supplementary Table 5 for results on bacteria and fungi associated to leaves and roots separately). Next to effects on microbial communities, individual bacterial and fungal amplicon sequence variants (ASVs) responded to land-use treatments, too, as suggested by DESeq2 analysis (Fig. 2). Mowing did not significantly affect single bacterial ASVs, but the fertilizer treatment that significantly affected the abundance of 119 ASVs. Bacteria of the families *Caulobacteraceae*, *Comamonadaceae*, *Rhizobiaceae*, *Sphingobacteriaceae*, *Spirosomaceae*, *Chitinophagaceae*, and *Sphingomonadaceae* were among the taxa that most frequently differed significantly between fertilized and unfertilized plants (the complete list of bacterial ASVs with significant differences in abundances in the fertilizer treatment is given in Supplementary Tables 6). In contrast to bacteria, more fungal ASVs were found to respond to mowing treatments (*n* = 53) than to fertilizer treatments (*n* = 30). Fungi of the family *Teratosphaeriaceae* most strongly differed between the treatments that differed in mowing frequency and fertilizer addition (the complete list of fungal ASVs with significant differences in abundances in the fertilizer treatment is given in Supplementary Tables 7 and 8).

### Effects of land-use and the plant-associated microbiome on plant phenotype and performance

**Figure 3:**
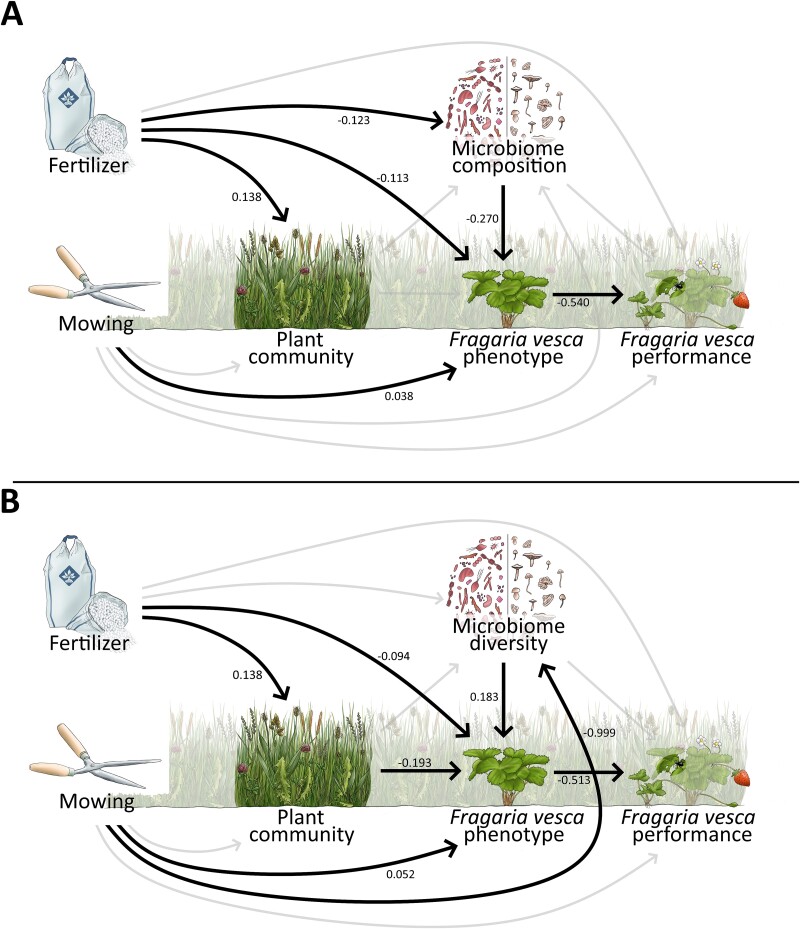
Effects of land-use components and microbiome on the phenotype and performance of *F. vesca* phytometers in a common garden experiment. Structural equation models (SEM) testing effects of land-use components, plant communities, and plant microbiome on the phenotype and performance of individual plants. One SEM is considering the microbiome composition (A), the other one the microbiome diversity (B). Numbers next to arrows represent standardized path coefficients and are given for significant paths only (bold arrows). Statistical results are reported in the text and supplementary material (Supplementary Tables 3 & 10–13).

We used structural equation modeling (SEM) to estimate the effect sizes of land-use components and plant microbiome on phenotype and performance of *F. vesca* plant individuals in a single directed analysis. The results of the analyses in the previous section serve as a validation of the chosen paths in building the structure of the model. For multivariate variables in the SEM, we used principal components (PC) as univariate representatives (morphological and physiological features of plant communities: PC1 = 45.4%; morphological and physiological features of *F. vesca* individuals: PC1 = 35.1%; performance of *F. vesca* individuals composed of herbivory, fruit set and flowers, vegetative reproduction: PC1 = 36.0%; microbiome composition: PC1 = 13.1%). We tested effects of microbial composition and diversity separately in two otherwise identical SEMs. Both structural equation models had a very good model fit (SEM1 (Fig. 3A): *P*_chi-square_ = 0.781; SEM2 (Fig. 3B): *P*_chi-square_ = 0.616; for further model fit parameters see Supplementary Table 9).

Overall, we found a clear dependence structure between the variables included in the SEMs. *F. vesca* performance, which showed no significant variation across land-use treatments (ANOVA; mowing: *F*_1,158_ = 1.98, *P* = 0.29; fertilizer: *F*_1,158_ = 2.51, *P* = 0.12), was solely affected by *F. vesca* phenotype in both SEMs (Fig. 3A, B), emphasizing the central role of the plants’ morphological and physiological traits. SEM results were supported by a clear correlation between phenotype and performance of *F. vesca* individuals (Mantel based on Pearson: *r* = 0.17, *P* = 0.006). The *F. vesca* phenotype, in turn, was directly affected by mowing and fertilizer (Fig. 3A, B), the morphological and physiological features of plant communities (Fig. 3B), and also by microbiome composition (Fig. 3A) and diversity (Fig. 3B).

In accordance with our second hypothesis, estimates of direct microbiome effects on *F. vesca* phenotype were stronger than those of the surrounding plant community and land-use components (Fig. 3A, B). However, note that the effect of plant communities was stronger than the effect of the diversity of microbial diversity (Fig. 3B). One morphological and four physiological traits of *Fragaria* individuals were individually related to differences in microbiome composition (Pearson correlation; leaf angle: *P* = 0.04, *r* = 0.23; greenness: *P* = <0.001, *r* = 0.31; hue: *P* = <0.001, *r* = 0.32; NDVI: *P* = <0.001, *r* = 0.31; PSRI: *P* = 0.007, *r* = −0.22). The multivariate *F. vesca* phenotype also corresponded to the multivariate representation of plant community features (Mantel: *r* = 0.15, *P* = 0.002). Some morphological and physiological traits of *F. vesca* and features of plant communities were directly correlated, too (Pearson correlation; leaf area: *P* = 0.002, *r* = 0.24; leaf area index: *P* < 0.001, *r* = 0.26; leaf area projected: *P* = 0.026, *r* = 0.18; leaf inclination: *P* = 0.003, *r* = −0.23; NPCI: *P* = 0.022, *r* = 0.18). Morphological and physiological features of plant communities as well as microbiome composition were affected by fertilizer addition (Fig. 3A, B), whereas microbiome diversity was affected by mowing treatments (Fig. 3B). Additional statistical analyses supported the Structural equation models: We found that mowing and the application for fertilization affected traits and performance of *F. vesca* (Supplementary Tables 3 and 10), the diversity and composition of bacterial and fungal communities (Supplementary Tables 4, 5, and10), community features (Supplementary Table 10). Additionally, the relationships between the factors of the SEM were supported (Supplementary Tables 11–13). In summary, these results verify the hypotheses that the plant microbiome has a stronger direct effect on the plant phenotype than direct land-use effects, and that plant performance is not a direct function of land-use intensity, supporting hypotheses two and three. Differences in single morphological and physiological features between the experimental land-use treatments could be found for both *Fragaria* phytometer plants as well as the surrounding plant communities (Supplementary Tables 2 & 4). In both individual plants and communities, fertilization led to an increased and denser growth as a result of the nutrient input, whereas mowing led to a reduction in biomass and height even after three months (but only considerably in the plant community, not the individuals). Both mowing and fertilization, and especially the combined mowing and fertilizer treatment, led to higher photosynthetic activity and lower levels of senescence (increased NDVI, decreased PSRI) on community level (Supplementary Fig. 1 and 2).

### Microbial effects on plant phenotype in the absence of other biotic or abiotic influences

**Figure 4:**
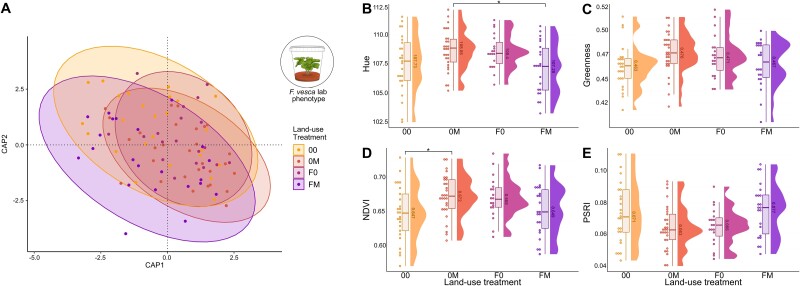
Phenotype of *F. Vesca* plants inoculated with the microbiome of field-grown phytometers that were exposed to different land-use treatments. *F. Vesca* Plants grew from surface-sterilized seeds in germ-free containers and experienced identical conditions until phenotyping; they only differed in their microbiome. Microbiome-mediated land-use treatment effects on (A) the multivariate *F. Vesca* phenotype or (B–E) individual physiological plant traits (hue, greenness, **n**ormalized **d**ifference **v**egetation **i**ndex, **p**lant **s**enescence **r**eflectance **i**ndex). Each circle represents one sample, land-use treatment is color-coded, significance is indicated with asterisks. Statistical results are reported in the text and supplementary material (Supplementary Table 13).

To test our fourth hypothesis that microbial effects are also measurable in the absence of other biotic of abiotic influences, we performed an additional lab experiment. We cultivated *F. vesca* plants from surface-sterilized seeds in germ-free containers and inoculated these plants with the whole microbiomes of the *F. vesca* phytometer plants retrieved from the field experiment. Thus, inoculated plants differed only in microbiome composition and diversity, but shared identical environmental condition. Inoculated plants were phenotyped three months after the inoculation using the plant scanner introduced above and the parameters describing morphological and physiological traits of *F. vesca* plants were used as dependent variable in the following statistical analysis. The multivariate phenotype of *F. vesca* clearly responded to microbiome origin, i.e. land-use effects on the microbiome of donor plants (field-grown phytometers) were visible in the phenotype of lab-grown plants that did not differ in any abiotic treatments but in microbiome composition and diversity (CAP based on Bray–Curtis distances; mowing: *F*_1,91_ = 0.75, *P* = 0.68; fertilizer: *F*_1,91_ = 0.52, *P* = 0.96; mowing × fertilizer: *F*_1,91_ = 1.99, *P* = 0.028; Fig. 4A).

Some physiological traits of lab-grown plants individually responded to land-use treatments (ANOVA of linear model with mowing × fertilizer; hue: *F*_1,91_ = 9.4, *P* = 0.003; greenness: *F*_1,91_ = 5.9, *P* = 0.017; NDVI: *F*_1,91_ = 8.9, *P* = 0.004; PSRI: *F*_1,91_ = 8.9, *P* = 0.004, Fig. 4B–E), which corresponds to those traits that directly responded to the microbiome in field-grown phytometer plants (see above). Responses of further plant traits are shown in Supplementary Fig. 3.

## Discussion

Our results largely verified our initial hypotheses on the prerequisites of microbiome-assisted plant tolerance against land-use changes. The microbiome of the *F. vesca* phytometers rapidly altered composition and diversity in response to mowing and fertilization. These alterations in the plant-associated microbiome directly affected the phytometers’ phenotype – an effect that was stronger than direct land-use effects on morphological and physiological traits of *F. vesca*. Note that the direct effect of plant communities (which may be an indirect effect of land-use treatment) is stronger than the effect of microbial diversity. As the performance of the plants was solely related to the plant phenotype and invariant across land-use treatments, we concluded that the plant microbiome may mediate land-use effects on plant phenotype and performance and thus may strongly contribute to plants’ tolerance to environmental changes. This conclusion was clearly supported by our laboratory experiments that demonstrated the microbiome’s potential to alter plant phenotypes in the absence of other biotic or abiotic influences associated with land-use changes. While our experiments clearly demonstrate that land-use treatment-specific microbiomes affect the phenotype of *F. vesca* plants, future studies may benefit from tracking shifts in the microbiome and associated changes in phenotype in response to land-use changes – as opposed to inoculating sterilized plants. In contrast to previous studies that transplanted microbiomes from sites with different levels of environmental stress to increase plant tolerance [9, 18–21], our data indicate the stress-mitigating potential of rapid modulations of the plant microbiome occurring on site. These natural modulations of the plant microbiome can be key in plant tolerance to global change components and thus a potential pathway in addition to “adapt or migrate” strategies [3] for species persistence and ecosystem stability.

Land-use intensification poses a major threat to many ecosystems, leading to species loss and alterations in community structure, which can disrupt ecosystem functioning [1, 2]. Relocation as a strategy to avoid environmental changes might often fail because of time constraints, continued habitat shrinking, or a lacking spatial connectivity within a species’ range of dispersal [73–76]; likewise, the process of evolutionary adaptation to rapid alterations also takes several generations and might be too slow in most cases [5]. Our data experimentally support recent findings that land-use not only affects plants and plant communities, but also the diversity and composition of plant-associated bacterial and fungal communities [11, 13, 15]. Our data additionally demonstrate the pace and small scale of such alterations: Within three months of exposure to different land-use treatments in the small area of the common garden, surrounded by the same environment, microbial communities clearly diverged to treatment-specific compositions. Thus, our data support the idea that alterations in the microbiome can easily keep pace with changes in environmental conditions [10]. These rapid alterations are the prerequisite for microbiome-assisted plant tolerance to changing environmental conditions, which has been proposed in transplantation studies [8, 9]. Our lab experiment provides important evidence—lacking in many other studies—of the contribution of the microbiome to the plant phenotype, which translates, according to our data, to plant performance. Therefore, small-scale natural variation in the plant microbiome in response to environmental conditions may be an untapped resource for microbiome engineering [23, 77] that aids ecosystem restoration [78].

Microorganisms play essential roles in ecosystem tolerance and resilience against global change components [79–81]. Our data indicate that rapid alterations of the plant microbiome may be key in plant species’ persistence despite changing environments and thus in ecosystem stability. However, a prerequisite for this microbiome-mediated strategy is locally available diverse sources of microorganisms that provide the source for rapid modulations in response to change. The need for a diverse selection of microbes is further supported by the finding that the strongest effects on plants are usually mediated by microbial consortia, not individual strains [35], and that microbial diversity can enhance plant species richness and productivity [14, 22]. Therefore, we conclude that conservation efforts will benefit from considering and protecting microbial diversity in addition to efforts to maintain plant and animal communities. Given the multiple beneficial roles of microbes in natural and anthropogenically altered environments, such “microbiome stewardship” [82] can help mitigate the effects of global change and facilitate environmental and human health.

## Supplementary Material

Zieschank_et_al_ISME_SupplementaryMaterial_R2_wraf010

## Data Availability

This work was performed within the scope and using resources of the project EXClAvE of the Biodiversity Exploratories program (DFG Priority Program 1374). The dataset generated and analyzed during the current study is publicly available in the Biodiversity Exploratories Information System (#31497, https://doi.org/10.25829/bexis.31497-13). The raw sequences of high-throughput 16S and ITS amplicon sequencing are available at the European Nucleotide Archive ENA under the project accession PRJEB52951 (https://www.ebi.ac.uk/ena/browser/view/PRJEB52951).
