# Supplementary material for: Tolerance to land-use changes through natural modulations of the plant microbiome
Source: ISME J. 2025 Jan 21;19(1):wraf010. doi: 10.1093/ismejo/wraf010 (PMC11833322; doi:10.1093/ismejo/wraf010)
Supplement: Zieschank_et_al_ISME_SupplementaryMaterial_R2_wraf010 [file zieschank_et_al_isme_supplementarymaterial_r2_wraf010.docx]

**Tolerance to land-use changes through natural modulations of the plant microbiome**

**Vincent Zieschank, Anne Muola, Stefan Janssen, Alexander Lach and Robert R. Junker**

**Supplementary Information**

**Supplementary Tables**

**Supplementary Table 1.** Land-use effects and effects of plot origin on composition of bacterial and fungal communities associated to leaves and roots of *Fragaria vesca* (grown in common garden). Results of distance-based redundancy analyses using Bray-Curtis distances followed by a permutation test under reduced model with subsequent analysis of variance are shown. Significant results are indicated in bold.

|  |  | Treatment | | Origin | | Treatment x Origin | |
| --- | --- | --- | --- | --- | --- | --- | --- |
|  | | F_3,138_ | *p* | F_2,138_ | *p* | F_6,138_ | *p* |
| *Fragaria vesca* phenotype | | 16.215 | **<0.001** | 1.259 | 0.287 | 0.767 | 0.597 |
| *Fragaria vesca* performance | | 1.712 | 0.167 | 0.556 | 0.575 | 1.047 | 0.398 |
| Microbiome composition | | 3.822 | **0.011** | 0.688 | 0.561 | 0.708 | 0.701 |
| Microbiome  diversity | | 2.475 | 0.064 | 0.360 | 0.782 | 0.621 | 0.778 |

**Supplementary Table 2.** Moran’s I for endogenous variables of both structural equation models (see Fig. 2A,B in the main text) to test for spatial autocorrelation. Significant results are indicated in bold.

| model | variable | *p* |
| --- | --- | --- |
| SEM 1 (Fig. 2A) | plant community | <0.001 |
|  | *F. vesca* phenotype | 0.1854 |
|  | *F. vesca* performance | 0.06792 |
|  | microbiome composition | 0.6586 |
| SEM 2 (Fig. 2B) | plant community | <0.001 |
|  | *F. vesca* phenotype | 0.1883 |
|  | *F. vesca* performance | 0.06679 |
|  | microbiome diversity | 0.9756 |

**Supplementary Table 3.** Land-use effects on morphological and physiological parameters of *F. vesca* phytometers grown in the common garden. Results of ANOVA with mowing and fertilizer as explanatory variables are shown. Significant results are indicated in bold.

| parameter | mowing | | fertilizer | | mowing x fertilizer | |
| --- | --- | --- | --- | --- | --- | --- |
|  | F_1,158_ | *p* | F_1,158_ | *p* | F_1,158_ | *p* |
| digital biomass | 3.860 | 0.051 | 45.020 | **<0.001** | 0.649 | 0.421 |
| height | 0.696 | 0.405 | 75.877 | **<0.001** | 0.901 | 0.344 |
| leaf angle | 0.832 | 0.363 | 6.393 | **0.012** | 0.001 | 0.975 |
| leaf area | 5.608 | **0.019** | 26.893 | **<0.001** | 2.687 | 0.103 |
| leaf area index | 4.087 | **0.045** | 23.951 | **<0.001** | 2.621 | 0.108 |
| leaf area projected | 4.195 | **0.042** | 16.438 | **<0.001** | 3.074 | 0.083 |
| leaf inclination | 1.824 | 0.179 | 21.330 | **<0.001** | 2.414 | 0.122 |
| light penetration depth | 0.414 | 0.521 | 19.683 | **<0.001** | 1.872 | 0.173 |
| greenness | 3.167 | 0.077 | 10.604 | **0.001** | 0.055 | 0.815 |
| hue | 0.031 | 0.860 | 0.110 | 0.741 | 0.521 | 0.472 |
| NDVI | 0.330 | 0.566 | 1.771 | 0.185 | 0.229 | 0.633 |
| NPCI | 1.251 | 0.265 | 1.474 | 0.227 | 1.606 | 0.207 |
| PSRI | 0.132 | 0.717 | 0.097 | 0.756 | 0.120 | 0.730 |

**Supplementary Table 4.** Land-use effects on bacterial and fungal diversity associated to leaves and roots of *Fragaria vesca* (grown in common garden). Results of ANOVA with mowing and fertilizer application explanatory variables are shown. Significant results are indicated in bold.

|  | sample | mowing | | fertilizer | |
| --- | --- | --- | --- | --- | --- |
|  |  | F_1,153_ | *p* | F_1,153_ | *p* |
| bacteria | leaf | 3.042 | 0.083 | 23.070 | **<0.001** |
|  | root | 0.407 | 0.524 | 0.490 | 0.485 |
| fungi | leaf | 9.501 | **0.002** | 0.785 | 0.377 |
|  | root | 1.890 | 0.171 | 4.046 | **0.046** |

**Supplementary Table 5.** Land-use effects on composition of bacterial and fungal communities associated to leaves and roots of *Fragaria vesca* (grown in common garden). Results of distance-based redundancy analyses using Bray-Curtis distances followed by a permutation test under reduced model with subsequent analysis of variance are shown. Significant results are indicated in bold.

|  | sample | mowing | | fertilizer | |
| --- | --- | --- | --- | --- | --- |
|  |  | F_1,153_ | *p* | F_1,153_ | *p* |
| bacteria | leaf | 1.641 | **0.007** | 4.615 | **<0.001** |
|  | root | 1.248 | **0.045** | 3.506 | **<0.001** |
| fungi | leaf | 3.572 | **<0.001** | 2.937 | **<0.001** |
|  | root | 1.206 | **0.044** | 1.861 | **<0.001** |

**Supplementary Table 6.** List of bacterial ASVs that significantly responded to fertilizer application that were identified using the R package DESeq2 (see Figure 4), sorted by effect size (log2FoldChange) to match the order in Figure 4 (left to right). Taxonomy was assigned using the q2‐feature‐classifier against the SILVA 138 99% OTUs reference sequences. Phyla are color-coded.

| **ASV_nr** | **log2FoldChange** | **Confidence** | **Domain** | **Phylum** | **Class** | **Order** | **Family** | **Genus** |
| --- | --- | --- | --- | --- | --- | --- | --- | --- |
| ASV_4214 | -5.122223285 | 0.996844831 | Bacteria | Verrucomicrobiota | Verrucomicrobiae | Pedosphaerales | Pedosphaeraceae | Ellin517 |
| ASV_10269 | -4.647600046 | 0.900880892 | Bacteria | Proteobacteria | Gammaproteobacteria | Pseudomonadales | Spongiibacteraceae | BD1-7_clade |
| ASV_10904 | -4.615689053 | 0.99989169 | Bacteria | Proteobacteria | Gammaproteobacteria | Burkholderiales | Nitrosomonadaceae | Ellin6067 |
| ASV_18569 | -4.603404318 | 0.796477938 | Bacteria | Bdellovibrionota | Oligoflexia | 0319-6G20 | 0319-6G20 | 0319-6G20 |
| ASV_1820 | -4.507807542 | 0.986777594 | Bacteria | Proteobacteria | Gammaproteobacteria | Burkholderiales | Gallionellaceae | NA |
| ASV_17008 | -4.22067128 | 0.912857887 | Bacteria | Proteobacteria | Alphaproteobacteria | Sphingomonadales | Sphingomonadaceae | Sphingobium |
| ASV_19304 | -4.14913106 | 0.964458721 | Bacteria | Myxococcota | Polyangia | Polyangiales | BIrii41 | BIrii41 |
| ASV_16583 | -4.093075966 | 0.999999759 | Bacteria | Bacteroidota | Bacteroidia | NA | NA | NA |
| ASV_2385 | -4.045807206 | 0.999267027 | Bacteria | Acidobacteriota | Acidobacteriae | Acidobacteriales | Acidobacteriaceae | Terriglobus |
| ASV_15034 | -4.011169971 | 0.996634271 | Bacteria | Proteobacteria | Alphaproteobacteria | Rhizobiales | Beijerinckiaceae | 1174-901-12 |
| ASV_2731 | -4.005801237 | 0.9979017 | Bacteria | Bacteroidota | Bacteroidia | Cytophagales | Hymenobacteraceae | Hymenobacter |
| ASV_19226 | -3.948437342 | 0.999998163 | Bacteria | Proteobacteria | Gammaproteobacteria | Burkholderiales | Comamonadaceae | NA |
| ASV_4037 | -3.931313284 | 0.999961991 | Bacteria | Bacteroidota | Bacteroidia | Sphingobacteriales | KD3-93 | KD3-93 |
| ASV_12668 | -3.819563884 | 0.973407984 | Bacteria | Proteobacteria | Alphaproteobacteria | Azospirillales | Azospirillaceae | uncultured |
| ASV_17568 | -3.702578382 | 0.999654991 | Bacteria | Bacteroidota | Bacteroidia | Chitinophagales | Chitinophagaceae | NA |
| ASV_11646 | -3.590305806 | 0.999981693 | Bacteria | Proteobacteria | Gammaproteobacteria | Pseudomonadales | NA | NA |
| ASV_105 | -3.085340138 | 0.997494028 | Bacteria | Proteobacteria | Alphaproteobacteria | Rhodospirillales | uncultured | uncultured |
| ASV_9462 | -2.875337971 | 0.984181691 | Bacteria | Bacteroidota | Bacteroidia | Cytophagales | Hymenobacteraceae | Hymenobacter |
| ASV_7707 | -2.835477685 | 0.999921217 | Bacteria | Proteobacteria | Gammaproteobacteria | Pseudomonadales | Spongiibacteraceae | BD1-7_clade |
| ASV_8711 | -2.805067884 | 0.999991756 | Bacteria | Planctomycetota | vadinHA49 | vadinHA49 | vadinHA49 | vadinHA49 |
| ASV_2649 | -2.74153524 | 0.99999823 | Bacteria | FCPU426 | FCPU426 | FCPU426 | FCPU426 | FCPU426 |
| ASV_12709 | -2.339591967 | 0.988020014 | Bacteria | Proteobacteria | Alphaproteobacteria | NA | NA | NA |
| ASV_12775 | -2.330390821 | 0.997645323 | Bacteria | Margulisbacteria | Margulisbacteria | Margulisbacteria | Margulisbacteria | Margulisbacteria |
| ASV_12336 | -2.297984022 | 0.989159166 | Bacteria | Bacteroidota | Bacteroidia | Chitinophagales | uncultured | uncultured |
| ASV_7206 | -2.19674238 | 0.999999836 | Bacteria | Bdellovibrionota | Bdellovibrionia | Bdellovibrionales | Bdellovibrionaceae | Bdellovibrio |
| ASV_9551 | -1.909581139 | 0.999999266 | Bacteria | Bacteroidota | Bacteroidia | Sphingobacteriales | AKYH767 | AKYH767 |
| ASV_9252 | -1.829383505 | 0.999823882 | Bacteria | Bacteroidota | Bacteroidia | Cytophagales | Hymenobacteraceae | Hymenobacter |
| ASV_16942 | -1.463031522 | 0.77308458 | Bacteria | Bacteroidota | Bacteroidia | Cytophagales | Microscillaceae | Ohtaekwangia |
| ASV_13256 | -1.277889449 | 0.999705568 | Bacteria | Bacteroidota | Bacteroidia | Cytophagales | Hymenobacteraceae | Hymenobacter |
| ASV_16985 | -1.275383831 | 0.737634778 | Bacteria | Proteobacteria | Alphaproteobacteria | Rhizobiales | Rhizobiaceae | NA |
| ASV_12800 | -1.242414349 | 0.974977221 | Bacteria | Gemmatimonadota | Gemmatimonadetes | Gemmatimonadales | Gemmatimonadaceae | uncultured |
| ASV_3565 | -1.160517817 | 0.878206281 | Bacteria | Proteobacteria | Alphaproteobacteria | Sphingomonadales | Sphingomonadaceae | Sphingomonas |
| ASV_15616 | -1.028269521 | 0.999966569 | Bacteria | Bacteroidota | Bacteroidia | Cytophagales | Spirosomaceae | NA |
| ASV_12371 | -1.013740082 | 0.970743401 | Bacteria | Bacteroidota | Bacteroidia | Sphingobacteriales | Sphingobacteriaceae | Mucilaginibacter |
| ASV_13704 | -0.973757476 | 0.968845409 | Bacteria | Proteobacteria | Alphaproteobacteria | Rhizobiales | Incertae_Sedis | Bauldia |
| ASV_436 | -0.936132158 | 0.96260897 | Bacteria | Proteobacteria | Alphaproteobacteria | Rhizobiales | Xanthobacteraceae | Rhodoplanes |
| ASV_991 | -0.846289282 | 0.972047233 | Bacteria | Proteobacteria | Alphaproteobacteria | Rhizobiales | Incertae_Sedis | Bauldia |
| ASV_2664 | -0.7600728 | 0.99999997 | Bacteria | Proteobacteria | Alphaproteobacteria | Sphingomonadales | Sphingomonadaceae | NA |
| ASV_7134 | -0.746238954 | 0.982338814 | Bacteria | Proteobacteria | Alphaproteobacteria | Sphingomonadales | Sphingomonadaceae | Sphingobium |
| ASV_361 | -0.717994609 | 0.938341682 | Bacteria | Proteobacteria | Alphaproteobacteria | Rhizobiales | Rhizobiaceae | Aureimonas |
| ASV_15906 | -0.628809834 | 0.963202082 | Bacteria | Spirochaetota | Spirochaetia | Spirochaetales | Spirochaetaceae | Spirochaeta_2 |
| ASV_18550 | 0.629245894 | 0.986198346 | Bacteria | Proteobacteria | Gammaproteobacteria | Burkholderiales | A21b | A21b |
| ASV_8881 | 0.750328039 | 0.999997546 | Bacteria | Proteobacteria | Alphaproteobacteria | Rhizobiales | Xanthobacteraceae | NA |
| ASV_6077 | 0.759476285 | 0.998320762 | Bacteria | Bacteroidota | Bacteroidia | Chitinophagales | Saprospiraceae | uncultured |
| ASV_14025 | 0.79168114 | 0.999882759 | Bacteria | Bacteroidota | Bacteroidia | Cytophagales | Spirosomaceae | Dyadobacter |
| ASV_18087 | 0.902634509 | 0.99927108 | Bacteria | Proteobacteria | Alphaproteobacteria | Micropepsales | Micropepsaceae | uncultured |
| ASV_6611 | 0.952064567 | 0.999998894 | Bacteria | Proteobacteria | Alphaproteobacteria | Acetobacterales | Acetobacteraceae | NA |
| ASV_8989 | 0.978984919 | 0.820561667 | Bacteria | Proteobacteria | Alphaproteobacteria | Sphingomonadales | Sphingomonadaceae | Sphingobium |
| ASV_2062 | 0.999851365 | 0.997016117 | Bacteria | Bacteroidota | Bacteroidia | Sphingobacteriales | Sphingobacteriaceae | Pedobacter |
| ASV_19255 | 1.009165865 | 0.999996229 | Bacteria | Proteobacteria | Alphaproteobacteria | Sphingomonadales | Sphingomonadaceae | NA |
| ASV_13271 | 1.02824753 | 0.999938656 | Bacteria | Bacteroidota | Bacteroidia | Chitinophagales | Chitinophagaceae | NA |
| ASV_2848 | 1.088733746 | 0.997517884 | Bacteria | Proteobacteria | Alphaproteobacteria | Caulobacterales | Caulobacteraceae | Brevundimonas |
| ASV_1667 | 1.143527422 | 0.997491573 | Bacteria | Proteobacteria | Gammaproteobacteria | Burkholderiales | Methylophilaceae | NA |
| ASV_249 | 1.174259906 | 0.986163316 | Bacteria | Myxococcota | Polyangia | Polyangiales | NA | NA |
| ASV_16844 | 1.199253268 | 0.917560542 | Bacteria | Actinobacteriota | Actinobacteria | Corynebacteriales | Nocardiaceae | Rhodococcus |
| ASV_6039 | 1.300629575 | 0.98699256 | Bacteria | Proteobacteria | Gammaproteobacteria | Xanthomonadales | Rhodanobacteraceae | Rhodanobacter |
| ASV_9128 | 1.330230977 | 0.990151135 | Bacteria | Proteobacteria | Gammaproteobacteria | Pseudomonadales | Pseudomonadaceae | Pseudomonas |
| ASV_5316 | 1.394330812 | 0.997011959 | Bacteria | Proteobacteria | Gammaproteobacteria | Burkholderiales | Alcaligenaceae | Pigmentiphaga |
| ASV_11121 | 1.406707206 | 0.989901707 | Bacteria | Proteobacteria | Gammaproteobacteria | Xanthomonadales | Rhodanobacteraceae | Rhodanobacter |
| ASV_4250 | 1.442569419 | 0.999999861 | Bacteria | Bacteroidota | Bacteroidia | Cytophagales | Cytophagaceae | Cytophaga |
| ASV_9464 | 1.527425336 | 0.996649388 | Bacteria | Bacteroidota | Bacteroidia | Sphingobacteriales | Sphingobacteriaceae | Pedobacter |
| ASV_19295 | 1.555178927 | 0.999999886 | Bacteria | Verrucomicrobiota | Verrucomicrobiae | Pedosphaerales | Pedosphaeraceae | NA |
| ASV_16863 | 1.603635274 | 0.772016211 | Bacteria | Bacteroidota | Bacteroidia | Bacteroidetes | NA | NA |
| ASV_18294 | 1.641601974 | 0.99781201 | Bacteria | Proteobacteria | Alphaproteobacteria | Rhizobiales | Devosiaceae | NA |
| ASV_5731 | 1.659400422 | 0.872259245 | Bacteria | Proteobacteria | Gammaproteobacteria | Xanthomonadales | Xanthomonadaceae | Pseudoxanthomonas |
| ASV_12950 | 1.724423788 | 0.896941378 | Bacteria | Proteobacteria | Gammaproteobacteria | Pseudomonadales | Moraxellaceae | uncultured |
| ASV_1025 | 1.767662178 | 0.937547696 | Bacteria | Proteobacteria | Alphaproteobacteria | Rhizobiales | Xanthobacteraceae | Tardiphaga |
| ASV_1197 | 1.801341646 | 0.953552574 | Bacteria | Actinobacteriota | Actinobacteria | Propionibacteriales | Nocardioidaceae | Nocardioides |
| ASV_4227 | 1.804433975 | 0.948400758 | Bacteria | Proteobacteria | Gammaproteobacteria | Xanthomonadales | Xanthomonadaceae | Xanthomonas |
| ASV_16797 | 1.811281099 | 0.999972728 | Bacteria | Proteobacteria | Alphaproteobacteria | Caulobacterales | Caulobacteraceae | Asticcacaulis |
| ASV_3872 | 1.838877461 | 0.940129925 | Bacteria | Proteobacteria | Gammaproteobacteria | Burkholderiales | Alcaligenaceae | Verticiella |
| ASV_8050 | 1.840292347 | 0.857137274 | Bacteria | Proteobacteria | Gammaproteobacteria | Xanthomonadales | Xanthomonadaceae | Stenotrophomonas |
| ASV_6370 | 1.85613612 | 0.994602287 | Bacteria | Bacteroidota | Bacteroidia | Chitinophagales | Chitinophagaceae | Aurantisolimonas |
| ASV_6251 | 1.973247727 | 0.790049659 | Bacteria | Bacteroidota | Bacteroidia | Chitinophagales | Chitinophagaceae | uncultured |
| ASV_18600 | 2.140131023 | 0.989720523 | Bacteria | Proteobacteria | Alphaproteobacteria | Rhizobiales | Rhizobiaceae | NA |
| ASV_9416 | 2.300280296 | 0.99999894 | Bacteria | Proteobacteria | Alphaproteobacteria | Rhodobacterales | Rhodobacteraceae | NA |
| ASV_8438 | 2.310620995 | 0.999999803 | Bacteria | Bacteroidota | Bacteroidia | Flavobacteriales | Weeksellaceae | NA |
| ASV_18798 | 2.382622717 | 0.998035318 | Bacteria | Bacteroidota | Bacteroidia | Chitinophagales | Saprospiraceae | uncultured |
| ASV_11580 | 2.480162542 | 0.99999893 | Bacteria | Abditibacteriota | Abditibacteria | Abditibacteriales | Abditibacteriaceae | Abditibacterium |
| ASV_3658 | 2.520675065 | 0.996095489 | Bacteria | Proteobacteria | Alphaproteobacteria | Caulobacterales | Caulobacteraceae | Brevundimonas |
| ASV_16769 | 2.526181333 | 0.807133557 | Bacteria | Bacteroidota | Bacteroidia | Flavobacteriales | Weeksellaceae | Weeksellaceae |
| ASV_17898 | 2.550600918 | 0.999717526 | Bacteria | Myxococcota | Polyangia | Haliangiales | Haliangiaceae | Haliangium |
| ASV_11006 | 2.557877665 | 0.899368377 | Bacteria | Bacteroidota | Bacteroidia | Sphingobacteriales | Sphingobacteriaceae | Pedobacter |
| ASV_5093 | 2.684059367 | 0.707315169 | Bacteria | Proteobacteria | Alphaproteobacteria | Rhizobiales | Devosiaceae | Devosia |
| ASV_15632 | 2.71034018 | 0.999982025 | Bacteria | Bacteroidota | Bacteroidia | Cytophagales | Spirosomaceae | Dyadobacter |
| ASV_4816 | 2.783318837 | 0.999999594 | Bacteria | Proteobacteria | Gammaproteobacteria | Burkholderiales | Comamonadaceae | NA |
| ASV_4321 | 2.832898923 | 0.997461901 | Bacteria | Proteobacteria | Alphaproteobacteria | Rhizobiales | Rhizobiaceae | NA |
| ASV_14718 | 2.937522543 | 0.999999176 | Bacteria | Proteobacteria | Gammaproteobacteria | Xanthomonadales | NA | NA |
| ASV_8352 | 3.037026135 | 0.999668247 | Bacteria | Actinobacteriota | Thermoleophilia | Solirubrobacterales | Solirubrobacteraceae | uncultured |
| ASV_2395 | 3.210354646 | 0.999977467 | Bacteria | Bacteroidota | Bacteroidia | Cytophagales | Spirosomaceae | Dyadobacter |
| ASV_18360 | 3.233494442 | 0.896750512 | Bacteria | Proteobacteria | Gammaproteobacteria | Burkholderiales | Comamonadaceae | Aquabacterium |
| ASV_14215 | 3.349543215 | 0.999988136 | Bacteria | Proteobacteria | Gammaproteobacteria | Burkholderiales | Alcaligenaceae | NA |
| ASV_8509 | 3.470350978 | 0.970461714 | Bacteria | Proteobacteria | Alphaproteobacteria | Rhizobiales | Xanthobacteraceae | Pseudolabrys |
| ASV_11626 | 3.560896719 | 0.995599944 | Bacteria | Bacteroidota | Bacteroidia | Flavobacteriales | Flavobacteriaceae | Flavobacterium |
| ASV_11659 | 3.667061855 | 0.999999504 | Bacteria | Proteobacteria | Alphaproteobacteria | Sphingomonadales | Sphingomonadaceae | NA |
| ASV_7235 | 3.769524507 | 0.999430149 | Bacteria | Proteobacteria | Alphaproteobacteria | Rhizobiales | Rhizobiaceae | NA |
| ASV_10491 | 3.788229744 | 0.998089505 | Bacteria | Proteobacteria | Gammaproteobacteria | NA | NA | NA |
| ASV_19070 | 3.797349362 | 0.882200372 | Bacteria | Proteobacteria | Gammaproteobacteria | Xanthomonadales | Xanthomonadaceae | Pseudoxanthomonas |
| ASV_7612 | 3.804937198 | 0.99407751 | Bacteria | Bacteroidota | Bacteroidia | Cytophagales | Spirosomaceae | Spirosoma |
| ASV_10849 | 4.093670604 | 0.999723619 | Bacteria | Bacteroidota | Bacteroidia | Flavobacteriales | Flavobacteriaceae | Flavobacterium |
| ASV_13525 | 4.156858303 | 0.985983047 | Bacteria | Proteobacteria | Alphaproteobacteria | Sphingomonadales | Sphingomonadaceae | Sphingoaurantiacus |
| ASV_7837 | 4.183879367 | 0.999995238 | Bacteria | Bacteroidota | Bacteroidia | Chitinophagales | Chitinophagaceae | Edaphobaculum |
| ASV_17621 | 4.492746775 | 0.987287881 | Bacteria | Bacteroidota | Bacteroidia | Sphingobacteriales | Sphingobacteriaceae | Pedobacter |
| ASV_14765 | 4.573139051 | 0.99999999 | Bacteria | Proteobacteria | Alphaproteobacteria | Caulobacterales | Caulobacteraceae | NA |
| ASV_11862 | 4.838288099 | 0.999999929 | Bacteria | Bdellovibrionota | Bdellovibrionia | Bdellovibrionales | Bdellovibrionaceae | Bdellovibrio |
| ASV_5445 | 4.896392727 | 0.990982952 | Bacteria | Proteobacteria | Alphaproteobacteria | Rhodospirillales | uncultured | uncultured |
| ASV_13264 | 4.961016915 | 0.99999861 | Bacteria | Proteobacteria | Gammaproteobacteria | Burkholderiales | Comamonadaceae | NA |
| ASV_3667 | 4.982876111 | 0.998628573 | Bacteria | Myxococcota | Polyangia | Haliangiales | Haliangiaceae | Haliangium |
| ASV_12793 | 4.990480439 | 0.999246484 | Bacteria | Bacteroidota | Bacteroidia | Flavobacteriales | Flavobacteriaceae | Flavobacterium |
| ASV_15814 | 5.047257515 | 0.99300828 | Bacteria | Proteobacteria | Alphaproteobacteria | Caulobacterales | Caulobacteraceae | Phenylobacterium |
| ASV_6459 | 5.202875083 | 0.998959794 | Bacteria | Bacteroidota | Bacteroidia | Chitinophagales | Chitinophagaceae | Edaphobaculum |
| ASV_5757 | 5.246670346 | 0.70894282 | Bacteria | Proteobacteria | Alphaproteobacteria | Rhodobacterales | Rhodobacteraceae | Rhodobacter |
| ASV_3021 | 5.32336413 | 0.993508771 | Bacteria | Desulfobacterota | Desulfuromonadia | Geobacterales | Geobacteraceae | Geobacter |
| ASV_17853 | 5.34196907 | 0.86025101 | Bacteria | Bacteroidota | Bacteroidia | Flavobacteriales | Weeksellaceae | Chryseobacterium |
| ASV_12214 | 5.431631066 | 0.981020361 | Bacteria | Bacteroidota | Bacteroidia | Chitinophagales | Chitinophagaceae | Ferruginibacter |
| ASV_15350 | 5.539618021 | 0.965886009 | Bacteria | Proteobacteria | Gammaproteobacteria | Burkholderiales | Comamonadaceae | Aquabacterium |
| ASV_11337 | 5.594813329 | 0.982684547 | Bacteria | Bacteroidota | Bacteroidia | Chitinophagales | Chitinophagaceae | Edaphobaculum |
| ASV_10587 | 5.840268783 | 0.715688851 | Bacteria | Proteobacteria | Gammaproteobacteria | Burkholderiales | Methylophilaceae | Methylotenera |
| ASV_2138 | 6.090553675 | 0.998872443 | Bacteria | Bacteroidota | Bacteroidia | Flavobacteriales | Weeksellaceae | Chryseobacterium |

**Supplementary Table 7.** List of fungal ASVs that significantly responded to mowing application that were identified using the R package DESeq2 (see Figure 4), sorted by effect size (log2FoldChange) to match the order in Figure 4 (left to right). Taxonomy was assigned using the classify‐sklearn naïve Bayes taxonomy classifier against the UNITE 97% Version 10.0 reference sequences. Phyla are color-coded.

| **ASV_nr** | **log2FoldChange** | **Confidence** | **Domain** | **Phylum** | **Class** | **Order** | **Family** | **Genus** |
| --- | --- | --- | --- | --- | --- | --- | --- | --- |
| ASV_1291 | -25.84482782 | 0.742320257 | Fungi | Ascomycota | Dothideomycetes | Pleosporales | Sporormiaceae | Sporormiella |
| ASV_7797 | -6.639106562 | 0.999971251 | Fungi | Ascomycota | Dothideomycetes | NA | NA | NA |
| ASV_2811 | -5.14238584 | 0.968515615 | Fungi | Ascomycota | Dothideomycetes | Capnodiales | Teratosphaeriaceae | Recurvomyces |
| ASV_7706 | -5.002364632 | 0.978059311 | Fungi | Ascomycota | Dothideomycetes | Capnodiales | Teratosphaeriaceae | Recurvomyces |
| ASV_761 | -4.723824011 | 0.974861625 | Fungi | Basidiomycota | Agaricomycetes | Atheliales | Pilodermataceae | Tretomyces |
| ASV_764 | -4.260088005 | 0.982585849 | Fungi | Ascomycota | Dothideomycetes | Capnodiales | Teratosphaeriaceae | Recurvomyces |
| ASV_4550 | -3.982609499 | 0.998710976 | Fungi | Ascomycota | Dothideomycetes | Capnodiales | Teratosphaeriaceae | Recurvomyces |
| ASV_5006 | -3.942789912 | 0.999977631 | Fungi | Ascomycota | Dothideomycetes | NA | NA | NA |
| ASV_2145 | -3.819244222 | 0.816146937 | Fungi | Ascomycota | Dothideomycetes | Pleosporales | Sporormiaceae | Sporormiella |
| ASV_8268 | -3.697135471 | 0.999902007 | Fungi | Ascomycota | Dothideomycetes | NA | NA | NA |
| ASV_4444 | -3.540577114 | 0.999304355 | Fungi | Ascomycota | Dothideomycetes | Capnodiales | Teratosphaeriaceae | Recurvomyces |
| ASV_5651 | -3.481539399 | 0.723834377 | Fungi | Ascomycota | Dothideomycetes | Pleosporales | Sporormiaceae | Sporormiella |
| ASV_5516 | -3.450771623 | 0.981738123 | Fungi | Ascomycota | Dothideomycetes | Pleosporales | Sporormiaceae | Sporormiella |
| ASV_207 | -3.252646702 | 0.998957548 | Fungi | Ascomycota | Dothideomycetes | Capnodiales | Teratosphaeriaceae | Recurvomyces |
| ASV_2483 | -3.232044115 | 0.999969316 | Fungi | Ascomycota | Dothideomycetes | NA | NA | NA |
| ASV_6293 | -2.926294314 | 0.845232877 | Fungi | Ascomycota | Dothideomycetes | Capnodiales | Teratosphaeriaceae | Recurvomyces |
| ASV_3216 | -2.726367941 | 0.999881909 | Fungi | Ascomycota | Dothideomycetes | NA | NA | NA |
| ASV_6950 | -2.68478591 | 0.999955867 | Fungi | Ascomycota | Dothideomycetes | NA | NA | NA |
| ASV_5320 | -2.442196766 | 0.999990396 | Fungi | Ascomycota | Dothideomycetes | NA | NA | NA |
| ASV_4604 | -2.418753633 | 0.819605412 | Fungi | Ascomycota | Dothideomycetes | Pleosporales | Sporormiaceae | Sporormiella |
| ASV_5790 | -2.322938307 | 0.987003597 | Fungi | Ascomycota | Dothideomycetes | Capnodiales | Teratosphaeriaceae | Recurvomyces |
| ASV_2516 | -2.300568154 | 0.809735114 | Fungi | Ascomycota | Dothideomycetes | Pleosporales | Sporormiaceae | Sporormiella |
| ASV_6295 | -2.177044002 | 0.991452773 | Fungi | Ascomycota | Dothideomycetes | Pleosporales | Sporormiaceae | Sporormiella |
| ASV_8018 | -2.176995247 | 0.985645101 | Fungi | Ascomycota | Dothideomycetes | Capnodiales | Teratosphaeriaceae | Recurvomyces |
| ASV_3256 | -2.094628972 | 0.866381132 | Fungi | Ascomycota | Dothideomycetes | Capnodiales | Teratosphaeriaceae | Recurvomyces |
| ASV_2716 | -2.015262431 | 0.767117348 | Fungi | Ascomycota | Dothideomycetes | Capnodiales | Teratosphaeriaceae | Recurvomyces |
| ASV_2435 | -1.951557292 | 0.727926998 | Fungi | Ascomycota | Dothideomycetes | Capnodiales | Teratosphaeriaceae | Recurvomyces |
| ASV_4408 | -1.699169365 | 0.866427249 | Fungi | Basidiomycota | Agaricomycetes | Atheliales | Pilodermataceae | Tretomyces |
| ASV_6702 | -1.659147743 | 0.854224742 | Fungi | Ascomycota | Dothideomycetes | Capnodiales | Teratosphaeriaceae | Recurvomyces |
| ASV_5093 | -1.513222473 | 0.836468904 | Fungi | Ascomycota | Dothideomycetes | Capnodiales | Teratosphaeriaceae | Recurvomyces |
| ASV_6627 | -1.47232698 | 0.749670068 | Fungi | Ascomycota | Dothideomycetes | NA | NA | NA |
| ASV_2860 | -1.392385411 | 0.99991709 | Fungi | Ascomycota | Dothideomycetes | Capnodiales | Teratosphaeriaceae | Recurvomyces |
| ASV_1113 | -1.287594718 | 0.889308992 | Fungi | Ascomycota | Dothideomycetes | Pleosporales | Sporormiaceae | Sporormiella |
| ASV_3595 | -1.133147853 | 0.805467884 | Fungi | Ascomycota | Dothideomycetes | Pleosporales | Sporormiaceae | Sporormiella |
| ASV_7565 | -1.070030864 | 0.998316976 | Fungi | Ascomycota | Dothideomycetes | Capnodiales | Teratosphaeriaceae | Recurvomyces |
| ASV_2305 | -0.906128676 | 0.737749333 | Fungi | Ascomycota | Dothideomycetes | Pleosporales | Sporormiaceae | Sporormiella |
| ASV_2774 | 1.389248343 | 0.999917829 | Fungi | Ascomycota | Dothideomycetes | NA | NA | NA |
| ASV_1360 | 1.60308603 | 0.916021026 | Fungi | Ascomycota | Dothideomycetes | Pleosporales | Sporormiaceae | Sporormiella |
| ASV_3168 | 1.887794803 | 0.885970886 | Fungi | Ascomycota | Dothideomycetes | Pleosporales | Sporormiaceae | Sporormiella |
| ASV_4251 | 1.909287047 | 0.996647954 | Fungi | Ascomycota | Dothideomycetes | Capnodiales | Teratosphaeriaceae | Recurvomyces |
| ASV_6179 | 1.921012904 | 0.978189797 | Fungi | Ascomycota | Dothideomycetes | Capnodiales | Teratosphaeriaceae | Recurvomyces |
| ASV_2175 | 3.491135962 | 0.982996827 | Fungi | Ascomycota | Dothideomycetes | Capnodiales | Teratosphaeriaceae | Recurvomyces |
| ASV_1770 | 25.83402662 | 0.985526115 | Fungi | Ascomycota | Dothideomycetes | Capnodiales | Teratosphaeriaceae | Recurvomyces |

**Supplementary Table 8.** List of fungal ASVs that significantly responded to fertilizer application that were identified using the R package DESeq2 (see Figure 4), sorted by effect size (log2FoldChange) to match the order in Figure 4 (left to right). Taxonomy was assigned using the classify‐sklearn naïve Bayes taxonomy classifier against the UNITE 97% Version 10.0 reference sequences. Phyla are color-coded.

| **ASV_nr** | **log2FoldChange** | **Confidence** | **Domain** | **phylum** | **Class** | **Order** | **Family** | **Genus** |
| --- | --- | --- | --- | --- | --- | --- | --- | --- |
| ASV_216 | -4.165313424 | 0.992913435 | Fungi | Ascomycota | Dothideomycetes | Capnodiales | Teratosphaeriaceae | Recurvomyces |
| ASV_7405 | -4.128486011 | 0.826947927 | Fungi | Basidiomycota | Agaricomycetes | NA | NA | NA |
| ASV_5705 | -3.082602838 | 0.955398625 | Fungi | Ascomycota | Dothideomycetes | Capnodiales | Teratosphaeriaceae | Recurvomyces |
| ASV_4408 | -2.587451726 | 0.866427249 | Fungi | Basidiomycota | Agaricomycetes | Atheliales | Pilodermataceae | Tretomyces |
| ASV_4358 | -2.582048276 | 0.870908078 | Fungi | Basidiomycota | Agaricomycetes | Atheliales | Pilodermataceae | Tretomyces |
| ASV_2516 | -2.448540895 | 0.809735114 | Fungi | Ascomycota | Dothideomycetes | Pleosporales | Sporormiaceae | Sporormiella |
| ASV_7562 | -2.433818955 | 0.738564589 | Fungi | Ascomycota | NA | NA | NA | NA |
| ASV_3038 | -1.122841714 | 0.772107987 | Fungi | Ascomycota | Dothideomycetes | Capnodiales | Teratosphaeriaceae | Recurvomyces |
| ASV_2305 | -0.959034927 | 0.737749333 | Fungi | Ascomycota | Dothideomycetes | Pleosporales | Sporormiaceae | Sporormiella |
| ASV_6851 | -0.831716769 | 0.913865118 | Fungi | Ascomycota | Dothideomycetes | Capnodiales | Teratosphaeriaceae | Recurvomyces |
| ASV_7083 | -0.725296664 | 0.976340679 | Fungi | Ascomycota | Dothideomycetes | Capnodiales | Teratosphaeriaceae | Recurvomyces |
| ASV_5512 | -0.704349043 | 0.717803036 | Fungi | Basidiomycota | Agaricomycetes | Agaricales | Cortinariaceae | Cortinarius |
| ASV_4846 | 0.819848719 | 0.768718701 | Fungi | Ascomycota | Dothideomycetes | Capnodiales | Teratosphaeriaceae | Recurvomyces |
| ASV_7293 | 2.016063935 | 0.790559976 | Fungi | Ascomycota | Dothideomycetes | Pleosporales | Sporormiaceae | Sporormiella |
| ASV_4800 | 2.587486167 | 0.984687792 | Fungi | Ascomycota | Dothideomycetes | Capnodiales | Teratosphaeriaceae | Recurvomyces |
| ASV_2212 | 2.591873353 | 0.991014289 | Fungi | Ascomycota | Dothideomycetes | Capnodiales | Teratosphaeriaceae | Recurvomyces |
| ASV_6403 | 3.222776373 | 0.976499989 | Fungi | Ascomycota | Dothideomycetes | Capnodiales | Teratosphaeriaceae | Recurvomyces |
| ASV_7099 | 3.319340131 | 0.993734839 | Fungi | Ascomycota | Dothideomycetes | Capnodiales | Teratosphaeriaceae | Recurvomyces |
| ASV_1620 | 3.32408639 | 0.974462321 | Fungi | Ascomycota | Dothideomycetes | Pleosporales | Sporormiaceae | Sporormiella |
| ASV_2811 | 3.363527672 | 0.968515615 | Fungi | Ascomycota | Dothideomycetes | Capnodiales | Teratosphaeriaceae | Recurvomyces |
| ASV_3490 | 4.030785257 | 0.851763044 | Fungi | Ascomycota | Dothideomycetes | NA | NA | NA |
| ASV_16 | 4.816674327 | 0.999932026 | Fungi | Ascomycota | Dothideomycetes | NA | NA | NA |

**Supplementary Table 9.** Model fit parameters of both structural equation models (see Fig. 2A, B in the main text). SEM 1 uses microbiome composition, SEM 2 microbiome diversity as parameter.

| model | parameter | value |
| --- | --- | --- |
| SEM 1 (Fig. 2A) | *p*_chi-square_ | 0.781 |
|  | CFI | 1 |
|  | TLI | 1.106 |
|  | RMSEA | 0 |
|  | SRMR | 0.004 |
| SEM 2 (Fig. 2B) | *p*_chi-square_ | 0.616 |
|  | CFI | 1 |
|  | TLI | 1.093 |
|  | RMSEA | 0 |
|  | SRMR | 0.007 |

**Supplementary Table 10.** Land-use treatment (mowing and fertilizer application) effects on plant community features, *F. vesca* phenotype (grown in common garden), microbiome composition, microbiome diversity, and *F. vesca* performance (grown in common garden). Results of distance-based redundancy analyses using Bray-Curtis distances followed by a permutation test under reduced model with subsequent analysis of variance (in the case of plant community, *F. vesca* phenotype, microbiome composition) or of ANOVA with mowing and fertilizer application as explanatory variables (in the case of microbiome diversity, and *F. vesca* performance) are shown. Significant results are indicated in bold.

| plant community features | F_1,158_ | *p* |
| --- | --- | --- |
| mowing | 7.87 | **<0.001** |
| fertilizer | 7.27 | **<0.001** |
| mowing x fertilizer | 196 | **0.030** |
| *F. vesca* phenotype |  |  |
| mowing | 1.15 | 0.271 |
| fertilizer | 10.53 | **<0.001** |
| mowing x fertilizer | 1.96 | **0.029** |
| microbiome composition |  |  |
| mowing | 1.73 | **<0.001** |
| fertilizer | 3.39 | **<0.001** |
| mowing x fertilizer | 1.04 | 0.256 |
| microbiome diversity |  |  |
| mowing | 7.68 | **0.006** |
| fertilizer | 0.92 | 0.338 |
| mowing x fertilizer | 0.58 | 0.447 |
| *F. vesca* performance |  |  |
| mowing | 1.98 | 0.290 |
| fertilizer | 2.51 | 0.120 |
| mowing x fertilizer | 0.06 | 0.811 |

**Supplementary Table 11.** Association between F*. vesca* (grown in the common garden) phenotype and performance or plant community features. Results of Mantel tests based on Pearson’s correlations (9999 permutations) calculated from Bray-Curtis distances are shown. Significant results are indicated in bold.

| matrix 1 | matrix 2 | r | *p* |
| --- | --- | --- | --- |
| *F. vesca* phenotype | *F. vesca* performance | 0.17 | **0.006** |
| *F. vesca* phenotype | plant community features | 0.15 | **0.002** |

**Supplementary Table 12.** Associations between *Fragaria vesca* phenotype (morphological and physiological parameters) and microbiome composition (PC1) and *Fragaria vesca* phenotype (morphological and physiological parameters) and plant community features (PC1). Results of Pearson’s correlation are shown. Significant results are indicated in bold.

| parameter | microbiome composition | | plant community features | |
| --- | --- | --- | --- | --- |
|  | R | *p* | R | *p* |
| digital biomass | 0.09 | 0.29 | 0.08 | 0.300 |
| height | 0.062 | 0.44 | 0.11 | 0.150 |
| leaf angle | 0.23 | **0.004** | -0.14 | **0.027** |
| leaf area | 0.09 | 0.25 | 0.24 | **0.002** |
| leaf area index | 0.12 | 0.14 | 0.26 | **<0.001** |
| leaf area projected | 0.12 | 0.14 | 0.18 | **0.026** |
| leaf inclination | -0.05 | 0.55 | -0.23 | **0.003** |
| light penetration depth | -0.05 | 0.53 | 0.05 | 0.540 |
| greenness | 0.31 | **<0.001** | -0.001 | 0.990 |
| hue | 0.32 | **<0.001** | 0.08 | 0.330 |
| NDVI | 0.31 | **<0.001** | 0.01 | 0.870 |
| NPCI | -0.0009 | 0.99 | 0.18 | **0.022** |
| PSRI | -0.22 | **0.007** | 0.03 | 0.670 |

**Supplementary Table 13.** Microbiome-mediated effects on morphological and physiological parameters of *F. vesca* plants grown in the lab under containment. Microbiomes originating from *F. vesca* plants growing in the common garden under different land-use treatments were inoculated on *F. vesca* plants in the lab. Results of ANOVA with mowing and fertilizer as explanatory variables are shown. Significant results are indicated in bold.

| parameter | mowing | | fertilizer | | mowing x fertilizer | |
| --- | --- | --- | --- | --- | --- | --- |
|  | F_1,91_ | *p* | F_1,91_ | *p* | F_1,91_ | *P* |
| digital biomass | 0.433 | 0.512 | 0.120 | 0.730 | 0.032 | 0.860 |
| height | 0.209 | 0.648 | 0.016 | 0.899 | 0.036 | 0.850 |
| leaf angle | 2.052 | 0.155 | 0.742 | 0.391 | 0.767 | 0.383 |
| leaf area | 0.362 | 0.549 | 0.196 | 0.659 | 0.004 | 0.951 |
| leaf area index | 0.270 | 0.604 | 0.221 | 0.639 | 0.004 | 0.950 |
| leaf area projected | 0.791 | 0.376 | 0.319 | 0.574 | 0.007 | 0.934 |
| leaf inclination | 1.527 | 0.220 | 0.840 | 0.362 | 0.713 | 0.401 |
| light penetration depth | 0.035 | 0.851 | 0.082 | 0.775 | 0.551 | 0.460 |
| greenness | 0.795 | 0.375 | 0.074 | 0.786 | 5.905 | **0.017** |
| hue | 0.004 | 0.952 | 0.690 | 0.409 | 9.443 | **0.003** |
| NDVI | 0.862 | 0.355 | 0.004 | 0.952 | 8.929 | **0.004** |
| NPCI | 0.047 | 0.830 | 0.171 | 0.680 | 2.787 | 0.098 |
| PSRI | 0.043 | 0.836 | 0.266 | 0.607 | 8.900 | **0.004** |

**Supplementary Figures**

**Fig. S1**


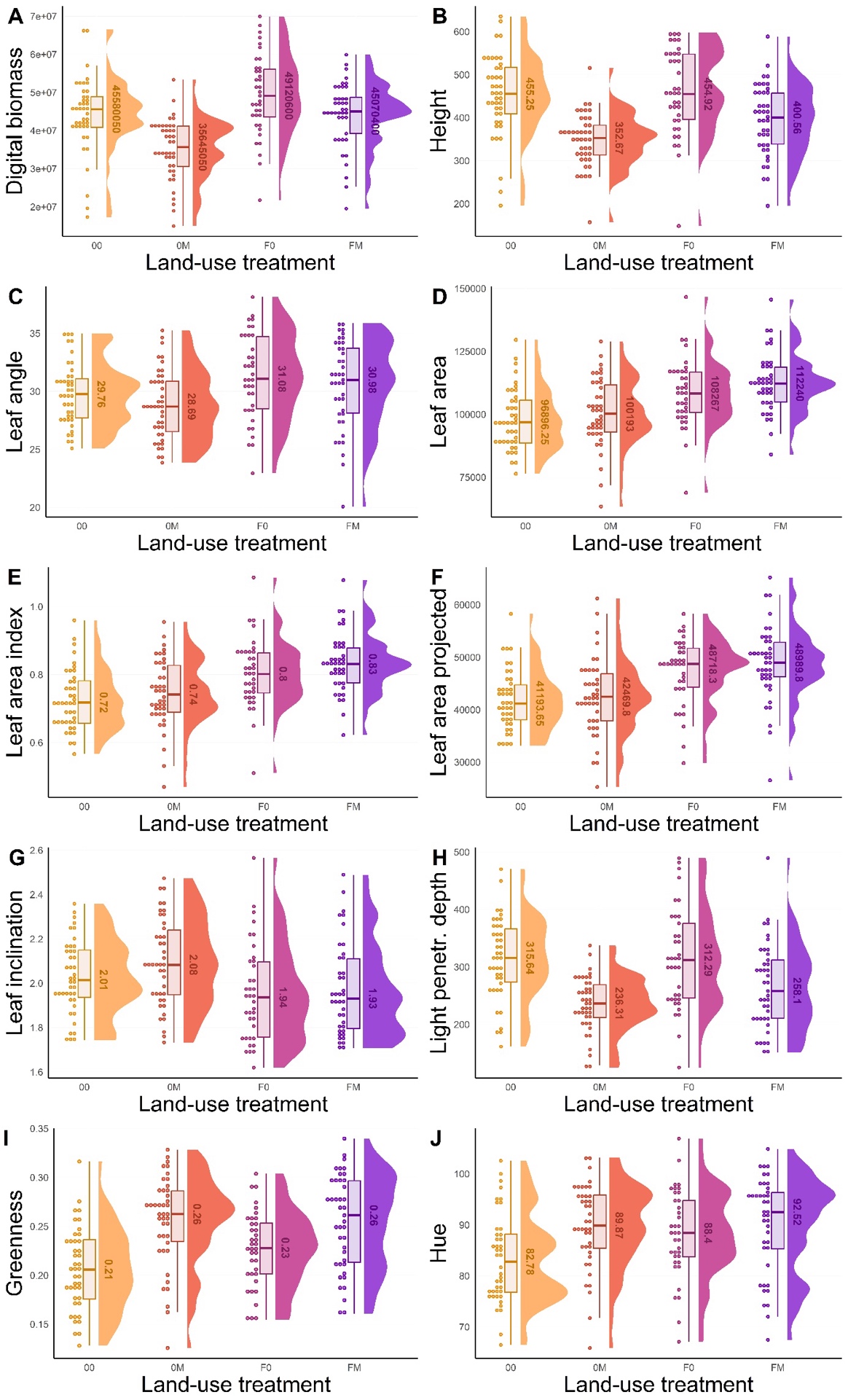


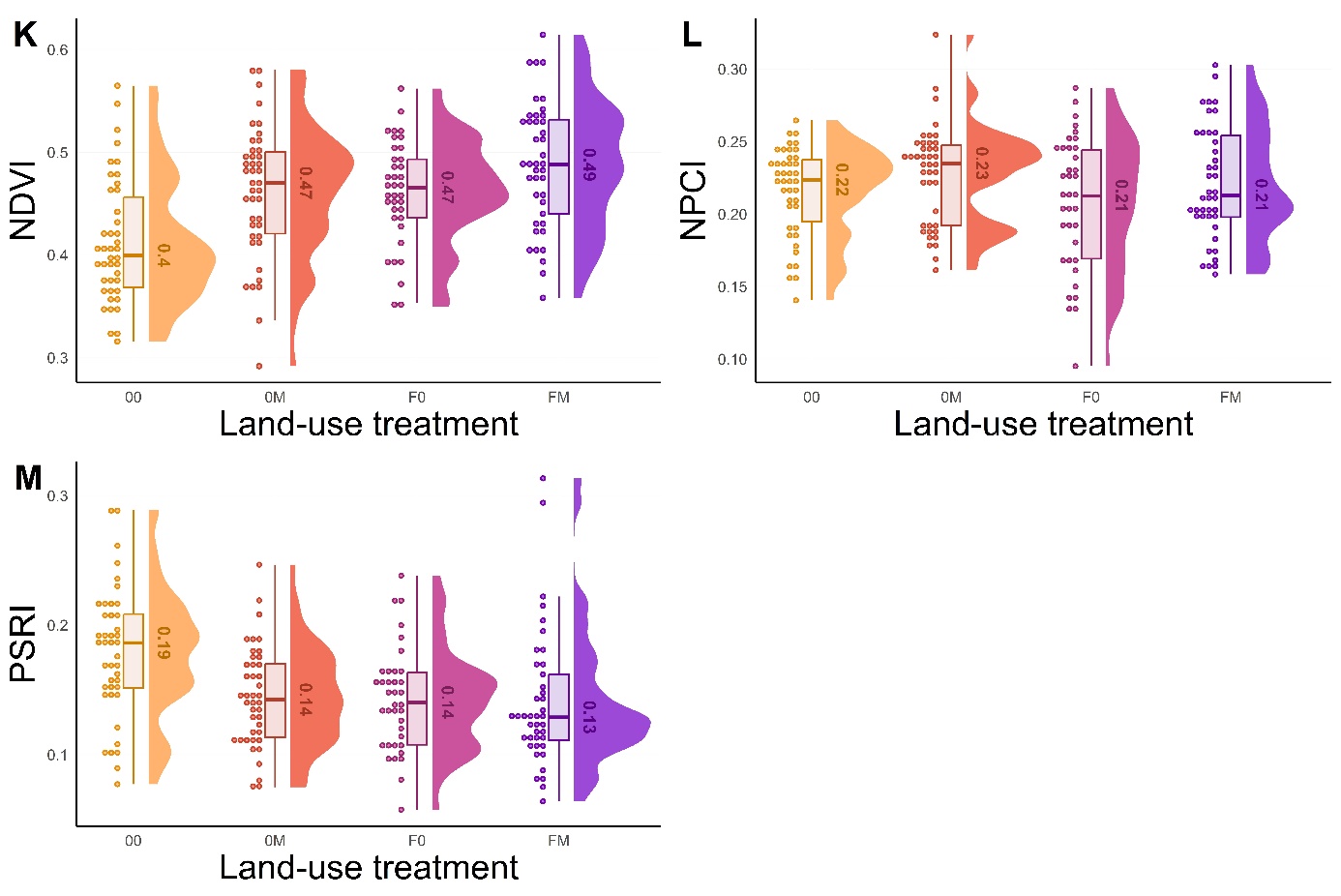


**Supplementary Figure 1. Community features of plant communities in the common garden that were exposed to different land-use treatments.** Land-use treatment effects on individual morphological (A-H) and physiological (I-M) plant community features (Digital biomass, Height, Leaf angle, Leaf area, Leaf area index, Leaf area projected, Leaf inclination, Light penetration depth, Greenness, Hue, **N**ormalized **D**ifference **V**egetation **I**ndex, **N**ormalized **P**igment **C**hlorophyll **I**ndex, **P**lant **S**enescence **R**eflectance **I**ndex). Each circle represents one sample and the land-use treatment is color-coded.

**Fig. S2**


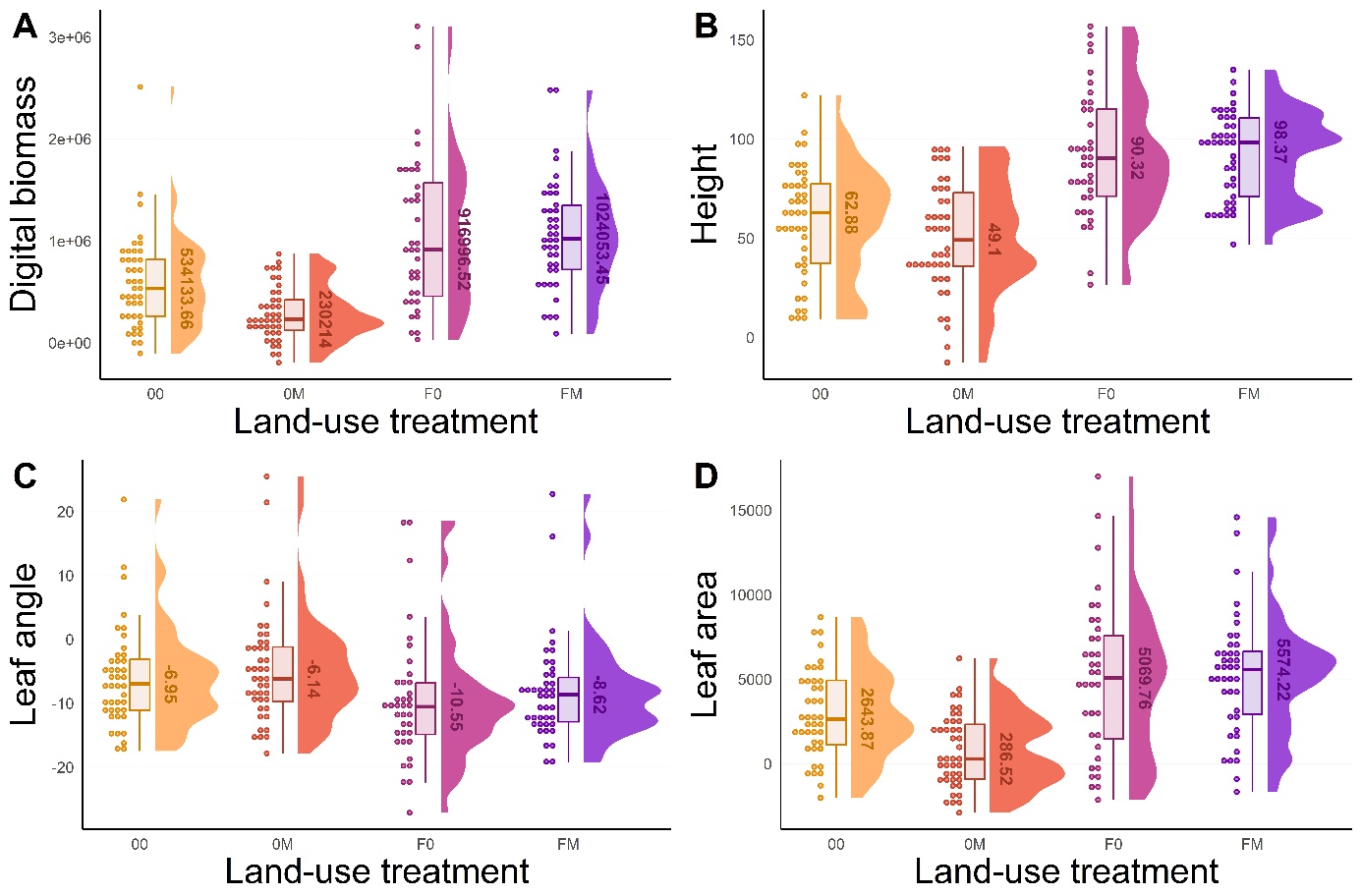


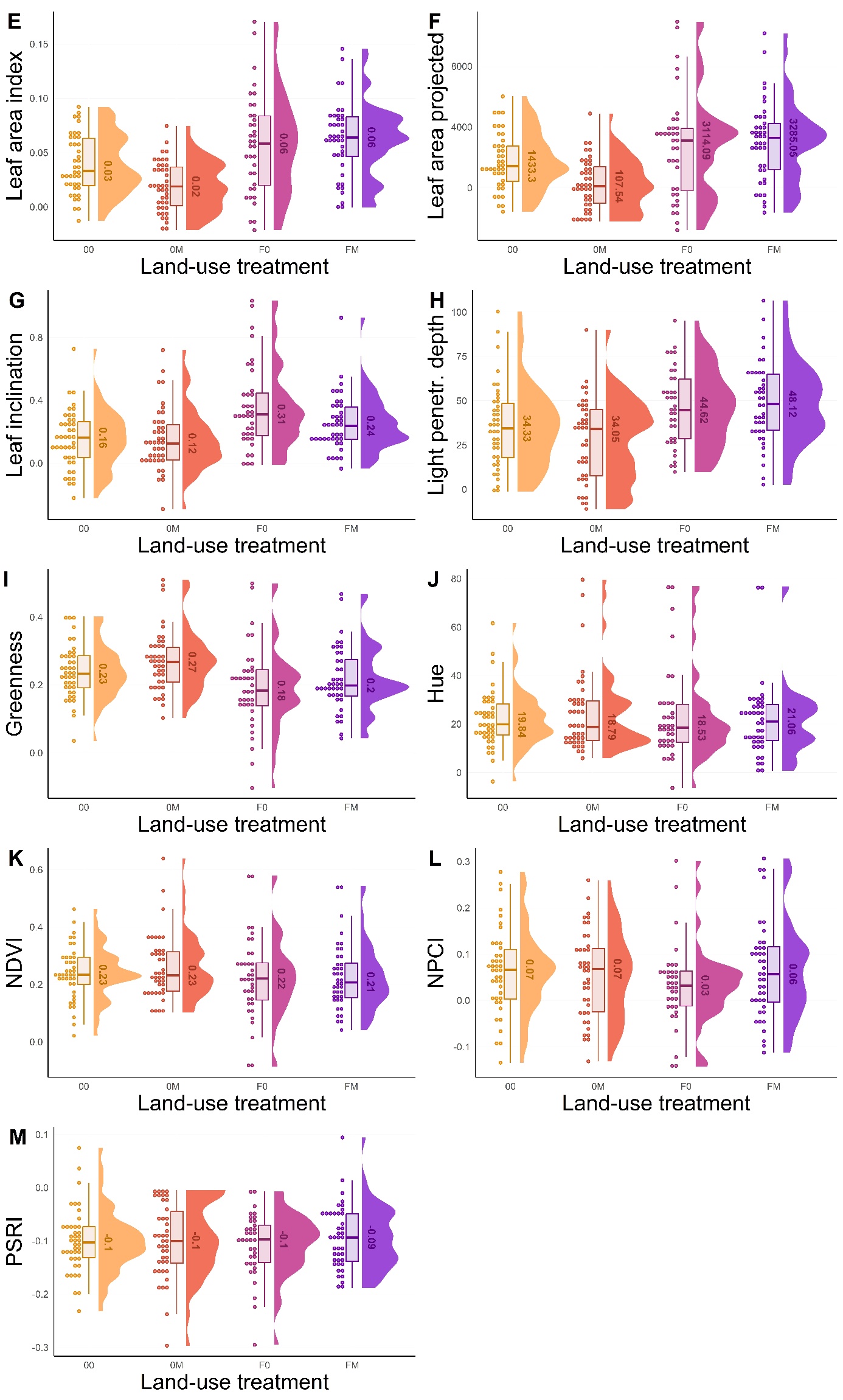


**Supplementary Figure 2. Phenotype of *F. vesca* plants that were growing within plant communities for 3 months while being exposed to different land-use treatments.** Land-use treatment effects on individual morphological (A-H) and physiological (I-M) plant traits (Digital biomass, Height, Leaf angle, Leaf area, Leaf area index, Leaf area projected, Leaf inclination, Light penetration depth, Greenness, Hue, **N**ormalized **D**ifference **V**egetation **I**ndex, **N**ormalized **P**igment **C**hlorophyll **I**ndex, **P**lant **S**enescence **R**eflectance **I**ndex). Each circle represents one sample and the land-use treatment is color-coded. Statistical results are reported in Supplementary Table 8.

**Fig. S3**


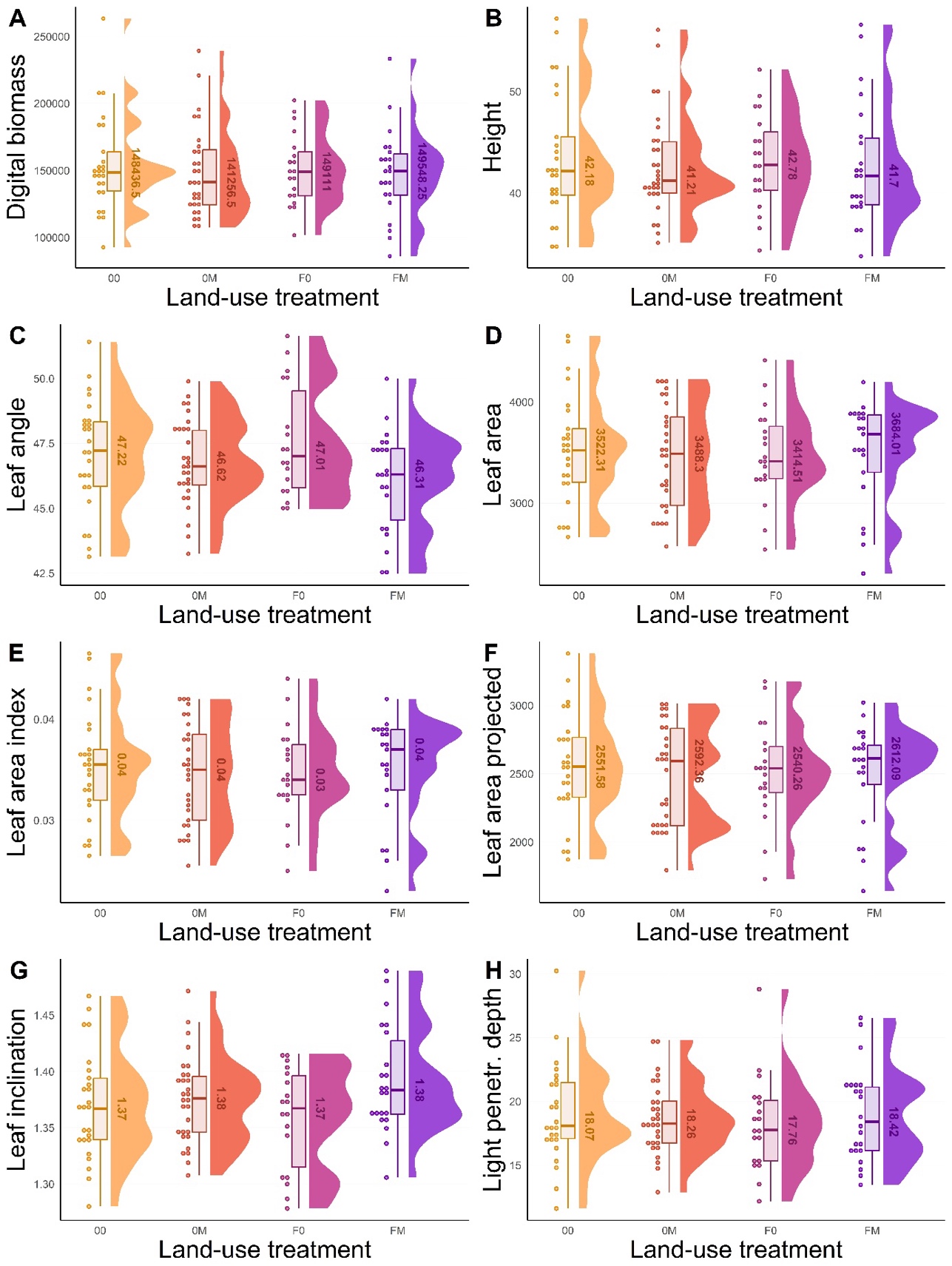


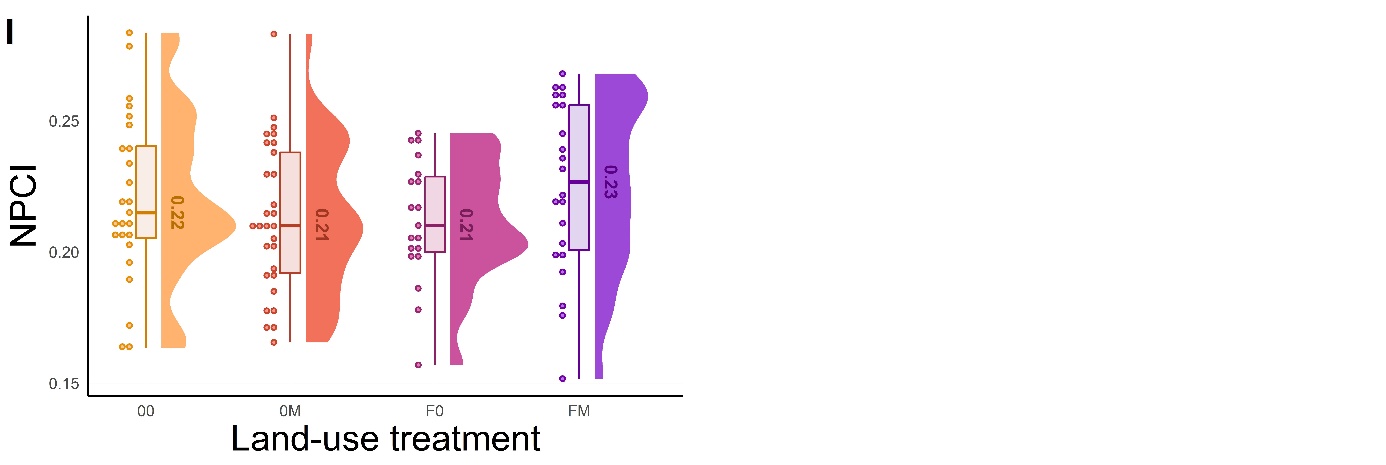


**Supplementary Figure 3. Phenotype of *F. vesca* plants inoculated with the microbiome of field-grown phytometers that were exposed to different land-use treatments.** *F. vesca* plants grew from surface-sterilized seeds in germ-free containers and experienced identical conditions until phenotyping; they only differed in their microbiome. Microbiome-mediated land-use treatment effects on individual morphological (A-H) and physiological (I) plant traits (Digital biomass, Height, Leaf angle, Leaf area, Leaf area index, Leaf area projected, Leaf inclination, Light penetration depth, **N**ormalized **P**igment **C**hlorophyll **I**ndex). Each circle represents one sample, land-use treatment is color-coded, significant parameters (Greenness, Hue, **N**ormalized **D**ifference **V**egetation **I**ndex, **P**lant **S**enescence **R**eflectance **I**ndex) are visualized in Figure 3. Statistical results are reported in Supplementary Table 9.
